# Enhancing Exosomal Delivery to Abdominal Aortic Aneurysms using Magnetically Responsive Chemotactic Nanomotors for Elastic Matrix Regenerative Repair

**DOI:** 10.1002/advs.202405085

**Published:** 2024-10-21

**Authors:** Lulu Wang, Yao Zhang, Chun Mao, Xiaoqiang Li

**Affiliations:** ^1^ Department of Vascular Surgery Nanjing Drum Tower Hospital Affiliated Hospital of Medical School Nanjing University Nanjing Jiangsu 210008 China; ^2^ National and Local Joint Engineering Research Center of Biomedical Functional Materials School of Chemistry and Materials Science Nanjing Normal University Nanjing Jiangsu 210023 China

**Keywords:** abdominal aortic aneurysms, elastic matrix regenerative repair, exosomal delivery, nanomotors, reversal

## Abstract

Abdominal aortic aneurysms (AAAs) involve localized dilation of the abdominal aorta, with the reversal of this condition being significantly limited by the inherently poor and abnormal regenerative repair of the aortic elastic matrix. Mesenchymal stem cell exosomes (MSCEs) are promising regenerative tools; however, achieving precise targeting of AAA with MSCEs is challenging owing to the high blood flow in the arterial system. In this study, an engineered exosomal nanomotor is developed for magnetic and chemical propulsion. The results demonstrate that this nanomotor effectively enhances the delivery of MSCEs to the AAA through magnetic field navigation and catalase‐induced chemotaxis. The nanomotor significantly enhances the elastic matrix repair, reduces oxidative stress, and activates the PI3K/Akt pathway, leading to aneurysm shrinkage and reversal. In addition, the nanomotor possesses magnetic resonance imaging capabilities. The use of this nanomotor offers a novel, targeted drug delivery system in a rat model of AAA and holds promise as a potential therapeutic option for this condition.

## Graphical abstract

MMS/MSCE/CAT is a dual‐driven nanomotor, guided by magnetic fields to accumulate in low‐flow regions near arterial walls and subsequently targets abdominal aortic aneurysms (AAAs) via chemotaxis. This improves the delivery efficiency of mesenchymal stem cell exosomes and promotes elastic matrix regeneration repair, reversing AAA progression through multiple mechanisms. Additionally, the nanomotor is MRI‐compatible.

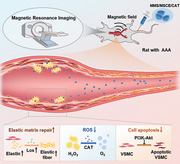

## 1 Introduction

Abdominal aortic aneurysm (AAA) is a common cardiovascular condition characterized by abnormal dilation of the abdominal aorta.^[^ ^1^ ^]^ It is highly prevalent and hazardous,^[^ ^2^ ^]^ and large AAAs often require surgical intervention. Currently, there is a lack of suitable treatment strategies for small AAAs, typically monitored until they reach a critical size necessitating surgery.^[^ ^3^ ^]^ A more proactive strategy involving pharmacological treatments to limit or reverse the progressive growth process of small AAAs shows promise,^[^ ^4^ ^]^ which is anticipated to delay or even eliminate the need for surgical intervention in patients. Implementing such a strategy could address the limitations of current treatment strategies.

A critical pathological feature of AAA is the disruption of the extracellular matrix (ECM). Unlike other ECM components of the vascular wall, elastic fibers, which bear the hemodynamic load of the arterial wall, cannot naturally regenerate or repair in adult blood vessels.^[^ ^5^ ^]^ Consequently, regenerating and repairing the elastic matrix in the vascular wall pose a significant challenge for effectively reversing small AAAs. Previous studies have shown that bone‐marrow‐derived mesenchymal stem cells (BM‐MSCs) can promote tissue repair and reverse AAA progression in animal models via paracrine effects on aneurysmal smooth muscle cells (SMCs).^[^ ^6^ ^]^ Exosomes, which play a crucial role in stem cell paracrine activity,^[^ ^7^ ^]^ mimic the therapeutic effects of donor cells,^[^ ^8^ ^]^ making exosome‐based cell‐free therapy a promising treatment for AAA. This approach also avoids complications such as immune response and tumor complications.^[^ ^9^ ^]^ However, the practical clinical application of exosomes is hindered by limitations such as insufficient targeting and susceptibility to rapid clearance.^[^ ^10^ ^]^ In addition, the high‐flow state in the aortic system^[^ ^11^ ^]^ further complicates the effective delivery of exosomes to the AAA vessel wall.

Engineered exosomes enhance targeting and prolong circulation,^[^ ^12^ ^]^ offering a viable delivery method for AAA treatment. However, traditional methods of engineering exosomes can damage their integrity and function.^[^ ^13^ ^]^ Using independently synthesized nanoscale artificial modules coupled with natural exosome modules can effectively protect the integrity of the membrane structure of exosomes and their biological activity while enhancing their functionality.^[^ ^14^ ^]^ Based on the hemodynamic characteristics of low flow velocity in the lumen of an AAA, where flow velocity decreases closer to the vascular wall,^[^ ^15^ ^]^ the artificial module was designed to move toward the wall. Specifically, this involved constructing a nanomotor artificial module with autonomous motion capabilities. The approach concentrates the engineered exosomes in the low‐flow region, mitigating systemic exosome metabolism caused by high‐velocity central blood flow. This can be achieved by equipping the nanomotor artificial module with magnetic targeting capabilities and achieving autonomous motion guided by an external magnetic field (MF).^[^ ^16^ ^]^ As a result, magnetic nanoparticles (MNPs) can overcome blood flow resistance to reach the AAA tube wall. Magnetic guidance has enabled the development of nanomedicine therapies for various cardiovascular diseases. For instance, MNPs guided by MF can accumulate in infarcted cardiac tissues, binding to damaged cardiomyocytes via anti‐MLC antibodies for treatment.^[^ ^17^ ^]^ Similarly, MNPs guided by MF can gather around arterial valves. This process helps anchor valvular interstitial cells with a hexapeptide ligand linked to PAR2, thus reducing aortic calcification.^[^ ^18^ ^]^ Given the continuous blood flow in the cardiovascular system, enhancing tissue anchoring after MNP enrichment is crucial. Compared with the specific binding of antibodies mentioned above, an innovative approach involves leveraging high levels of hydrogen peroxide (H_2_O_2_) in the AAA microenvironment for anchoring.^[^ ^19^ ^]^ Catalase (CAT)‐catalyzed H_2_O_2_ breakdown propels nanomotors,^[^ ^20^ ^]^ enabling chemotaxis toward the AAA and facilitating secondary targeting.

**Figure 1:**
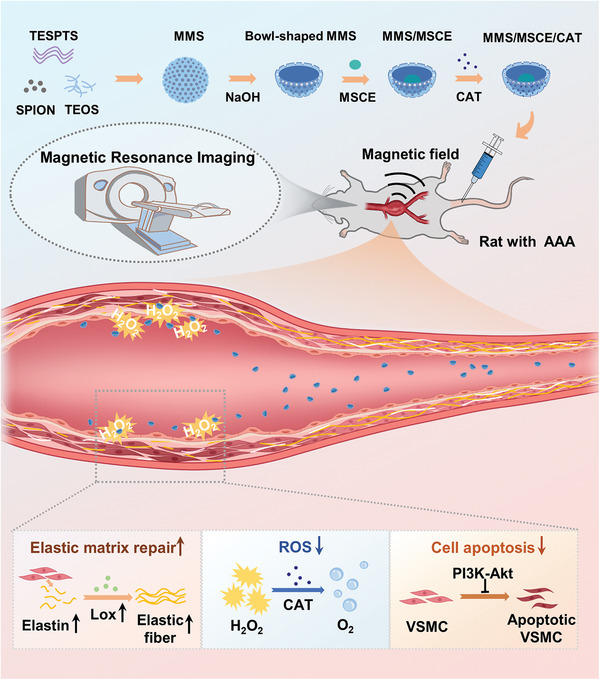
Schematic illustrating the fabrication of MMS/MSCE/CAT nanomotors and their magnetically guided enrichment in the vascular wall, chemotaxis to the disease microenvironment, treatment of AAA, and enhanced MRI of AAA.

Based on this approach, we developed an effective exosome nanodelivery platform with dual targeting via magnetic guidance and CAT‐induced chemotaxis. This platform facilitates the targeted delivery of mesenchymal stem cell exosomes (MSCEs) in AAA for regenerative repair of the elastic matrix (**Figure** **1**). To achieve MF‐guided nanomotor enrichment in AAA tubule walls, we magnetized mesoporous silica by embedding superparamagnetic iron oxide nanoparticles (SPION) into its core. Then, we shaped the resulting material into a bowl structure by etching for structural asymmetry, enhancing directional movement.^[^ ^21^ ^]^ Covalent crosslinking was used to couple the MSCE to the nanomotor. Additionally, loading mesopores with CAT enables chemotactic nanomotor movement in high H_2_O_2_ environments. In the AAA rat model, upon tail vein injection, the nanomotors were guided through MF to migrate from the high‐ to low‐flow zones, followed by chemotactic targeting induced by the high H_2_O_2_ environment at the vessel walls. Nanomotor uptake by vascular smooth muscle cells (VSMCs) stimulates elastic matrix regenerative repair, reduces reactive oxygen species (ROS), activates PI3K/Akt signaling, and modulates apoptosis, leading to a significant reduction in aneurysm diameter and reversal of AAA progression. Furthermore, the magnetic resonance imaging (MRI) capability of the SPION allows the nanomotor to serve as a potential T_2_ MRI contrast agent.^[^ ^22^ ^]^ This nanoplatform leverages the specific characteristics of the AAA environment to enable a graded targeted delivery strategy, enhancing the efficacy of exosomes in targeting AAA within complex blood environments.

## 2 Results and Discussion

### 2.1 Preparation and Characterization of the Nanomotors

Natural exosomal vesicles were isolated from BM‐MSCs via ultracentrifugation.^[^ ^23^ ^]^ The results of transmission electron microscopy revealed that the MSCEs were round or oval membranous vesicles, ≈30.0–150.0 nm in diameter (Figure S1A, Supporting Information). The nanoparticle tracking analysis results showed that the mean diameter of exosomes was 92.9 nm with a concentration of 5.1 × 10^7^ particles mL^−1^ (Figure S1B, Supporting Information). Western blot (WB) analysis confirmed the presence of common exosome markers, such as TSG101 and CD63, in the isolated vesicles. Moreover, the exosomes were also positive for mesenchymal stem cell (MSC) markers, including CD73 and vimentin, indicating that the exosomes were derived from MSCs as described (Figure S1C, Supporting Information).

**Figure 2:**
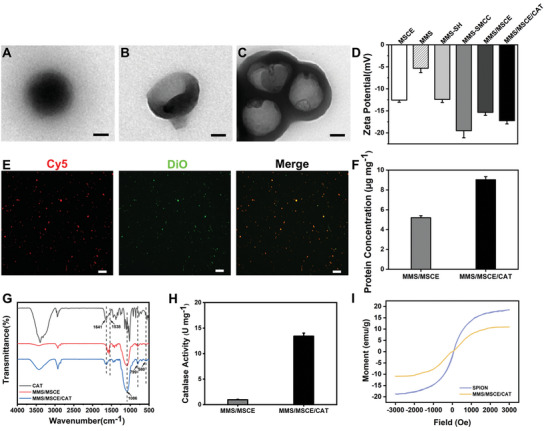
Characterization of different samples. Transmission electron microscopy images: A) Magnetic mesoporous silica nanoparticles (MMS); B) Bowl‐shaped MMS; C) MMS/MSCE/CAT. Scale bars = 50 nm. D) Zeta potential changes during the preparation process of the nanomotors. E) Confocal laser scanning microscope (CLSM) image of the nanomotors coupled with MSCE. Red, Cy5 labeled MMS; Green, DiO labeled MSCE; Scale bars = 10 µm. F) Protein concentration of MMS/MSCE and MMS/MSCE/CAT. G) Fourier‐transform infrared spectra of samples with different surface functional groups after catalase modification. H) Catalase activity of nanomotors at room temperature. I) Magnetic hysteresis loop for SPION and MMS/MSCE/CAT at room temperature. Experimental data are mean ± SD of samples in a representative experiment (*n* = 3).

Following a previously established method, spherical silica nanoparticles, ≈170.0 nm in size, were synthesized by incorporating organic and inorganic silicon encapsulated with SPION (**Figure** **2**A). Etching with NaOH selectively dissolved the inorganic silicon, resulting in contraction and deformation of the flexible organosilicon skeleton into asymmetric concave bowl‐shaped nanoparticles, which were also ≈170.0 nm in size (Figure 2B). It has been demonstrated that bowl‐shaped microcapsules are more easily internalized by SMCs compared to spherical microcapsules,^[^ ^24^ ^]^ in addition to the fact that this asymmetric geometry facilitates directional movement.^[^ ^21^ ^]^ Furthermore, they exhibit greater flexibility and deformability in solution,^[^ ^25^ ^]^ potentially facilitating coupling with MSCEs. The MMS was modified with (3‐Mercaptopropyl) trimethoxysilane to graft sulfhydryl groups onto its surface (denoted as MMS‐SH). The maleimide groups of the cross‐linker, cyclohexane‐1‐carboxylic acid 3‐sulfo‐N‐hydroxysuccinimide ester sodium salt (sulfo‐SMCC), reacted with the sulfhydryl groups on the surface of MMS‐SH to obtain MMS‐SMCC, which was further reacted with the amino groups on the surface of MSCE using NHS ester to form MMS/MSCE. After CAT loading, MMS/MSCE/CAT can be formed (Figure 2C). The surface functionalization process was characterized using zeta potential measurements (Figure 2D). After CAT conjugation, the abundant carboxyl groups in CAT resulted in a zeta potential of ≈ −17.2 mV for MMS/MSCE/CAT.

The coupling of the two modules was evaluated by fluorescence microscopy, which revealed the co‐localization of green fluorescence from DiO‐labeled MSCE with red fluorescence from Cy5‐labeled MMS, confirming successful coupling (Figure 2E). WB analysis confirmed the presence of MSCE markers in MMS/MSCE/CAT, again demonstrating successful loading of exosomes (Figure S1C, Supporting Information). The protein loading of MSCE and CAT was determined using the BCA kit. The MSCE protein content in MMS/MSCE and MMS/MSCE/CAT was ≈5.2 µg mg^−1^, and the CAT protein content in MMS/MSCE/CAT was ≈3.8 µg mg^−1^, i.e., the difference in protein amounts between MMS/MSCE and MMS/MSCE/CAT (Figure 2F). The linkage of CAT was further validated using Fourier‐transform infrared spectroscopy (Figure 2G). The spectrum of MMS/MSCE/CAT exhibits the Si‐O‐Si antisymmetric stretching vibration peak near 1086.0 cm^−1^, the symmetric stretching vibration peak of the Si‐O bond at 799.0 cm^−1^, and the Fe‐O‐Fe bond peak at 580.0 cm^−1^. Notably, compared to MMS/MSCE, distinctive peaks emerged at 1641.0 cm^−1^ and 1538.0 cm^−1^, corresponding to the amide (‐NH‐C = O‐) stretching vibration of the amino group, indicative of the presence of CAT.^[^ ^26^ ^]^ The enzymatic activity of CAT post‐loading onto the nanomotors was assessed, and the results revealed detectable CAT activity in MMS/MSCE/CAT samples relative to MMS/MSCE (Figure S2, Supporting Information), which was quantified to ≈13.4 U per mg of MMS/MSCE/CAT (Figure 2H). SPION may provide desired magnetic properties to MMS/MSCE/CAT. Consequently, MMS/MSCE/CAT, uniformly dispersed in phosphate buffer saline (PBS), could be drawn toward the tube wall under the influence of an MF, rendering the PBS solution clear again (Figure S3, Supporting Information). The hysteresis loop confirmed the magnetic efficacy of MMS/MSCE/CAT, exhibiting a saturation magnetic strength of 10.9 emu g^−1^ (Figure 2I).

### 2.2 Movement Behavior and Chemotactic Performance of the Nanomotors

In chemical field‐driven nanomotors, enzymes effectively facilitate self‐propulsion by converting in situ chemical energy into mechanical energy through catalysis. This motion was oriented toward regions of high substrate concentration, demonstrating chemotactic behavior.^[^ ^20^ ^]^ Previous studies indicated the presence of elevated H_2_O_2_ concentrations in the arterial walls of AAA,^[^ ^19^ ^]^ which could provide an enriched substrate environment for nanomotors. To emulate the AAA phenotype in vitro, primary SMCs from elastase‐induced AAA rat models (EaRASMCs) were initially extracted and validated (Figure S4, Supporting Information). Subsequently, EaRASMCs were stimulated with 10 ng mL^−1^ tumor necrosis factor (TNF)‐α and 10 ng mL^−1^ interleukin‐1β (IL‐1β) to induce the AAA phenotype, as described in a previous study.^[^ ^8^ ^]^ The concentration of H_2_O_2_ in the cells was assessed at different time points post‐stimulation, revealing the peak concentration of H_2_O_2_ (13.4 µM) after 24 h of stimulation (Figure S5, Supporting Information).

**Figure 3:**
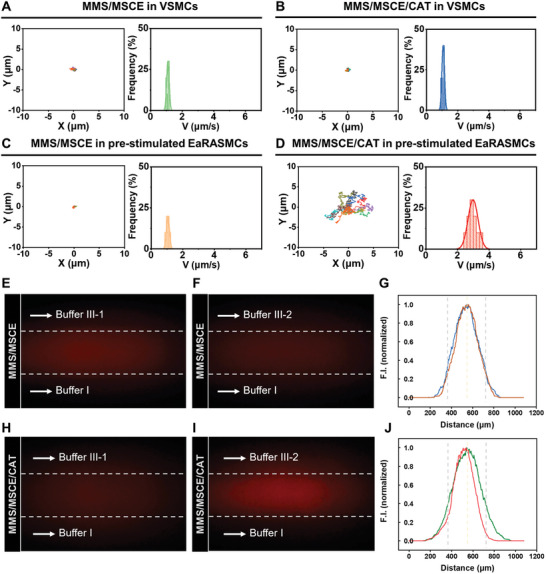
Trajectory and velocity distribution of A) MMS/MSCE and B) MMS/MSCE/CAT in VSMCs. Trajectory and velocity distribution of C) MMS/MSCE and D) MMS/MSCE/CAT in pre‐stimulated EaRASMCs (10 s, *n* = 10). Representative fluorescence images of MMS/MSCE flow in the presence of E) buffer I and buffer III‐1, and F) buffer I and buffer III‐2 on two sides, and G) their corresponding normalized fluorescence intensities. Representative fluorescence images of MMS/MSCE/CAT flow in the presence of H) buffer I and buffer III‐1, and I) buffer I and buffer III‐2 on both sides, J) and their corresponding normalized fluorescence intensities.

The motor behavior of MMS/MSCE and MMS/MSCE/CAT was examined within the cellular milieu of VSMCs and pre‐stimulated EaRASMCs. In the context of normal VSMCs, both MMS/MSCE and MMS/MSCE/CAT manifested Brownian motions, with average velocity distributions of 0.9–1.2 µm s^−1^ (**Figure** **3**A,B; Movies S1 and S2, Supporting Information). This behavior can be attributed to low levels of H_2_O_2_. However, after pre‐stimulating EaRASMCs with 10 ng mL^−1^ TNF‐α and 10 ng mL^−1^ IL‐1β for 24 h, significant changes were observed. The MMS/MSCE/CAT exhibited notable trajectories in different directions and increased velocity distributions, with the average motion velocity distribution escalating to 2.5–3.5 µm s^−1^. In contrast, the MMS/MSCE retained their Brownian motion characteristics (Figure 3C,D; Movies S3 and S4, Supporting Information).

The chemotaxis behavior of nanomotors within an in vivo flow environment was further examined using the classical Ψ‐shaped microfluidic channel model containing three inlets and one outlet (Figure S6, Supporting Information). Subsequently, the chemotaxis behavior of Cy5‐labeled MMS/MSCE/CAT was evaluated in a cellular milieu containing elevated concentrations of H_2_O_2_. The Cy5‐labeled MMS/MSCE was used as a comparative control. Buffers I, II, III‐1, and III‐2 comprised normal VSMCs lysates, Cy5‐labeled samples, normal VSMCs lysates, and pre‐stimulated EaRASMC lysates (possessing high H_2_O_2_ expression and serving as a chemotaxis inducer), respectively. The distribution of the fluorescently labeled samples dispersed in buffer II was evaluated via CLSM at a distance of 22 mm from the inlet. Upon introducing Cy5‐labeled MMS/MSCE into buffer II, the observed red fluorescence remained stationary, irrespective of whether buffer I and buffer III‐1 or buffer I and buffer III‐2 were adjacent (Figure 3E–G; Figure S7A,B, Supporting Information). This lack of movement was expected, as MMS/MSCE lacked inherent motility. In contrast, upon introducing Cy5‐labeled MMS/MSCE/CAT into buffer II, the central red fluorescence, indicative of Cy5‐labeled MMS/MSCE/CAT, exhibited negligible displacement in the absence of a chemical elicitor on both sides (Figure 3H; Figure S7C, Supporting Information). However, in the presence of a chemical elicitor on one side, the Cy5‐labeled MMS/MSCE/CAT significantly migrated toward buffer III‐2, traversing ≈21.0 µm (Figure 3I,J; Figure S7D, Supporting Information). These findings highlight the pronounced chemotactic response of the MMS/MSCE/CAT nanomotors in environments with elevated H_2_O_2_ levels under fluidic conditions, indicating their potential utility in AAA‐phenotyped EaRASMCs.

### 2.3 In Vitro Protection Effect of the Nanomotors on AAA

**Figure 4:**
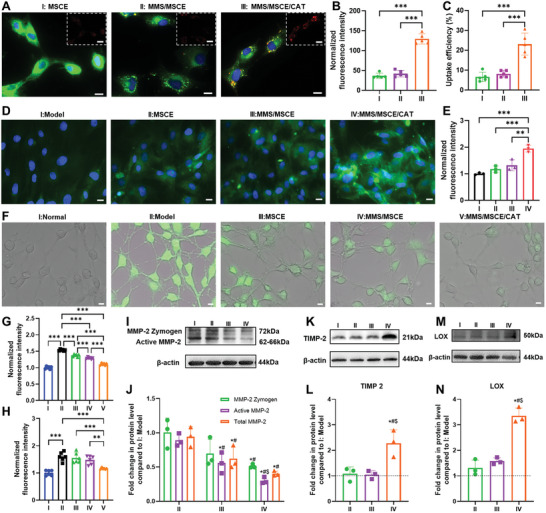
Elastic matrix regenerative repair, antioxidative stress, and anti‐proteolytic capability of MMS/MSCE/CAT in vitro. A,B) Fluorescence images and corresponding normalized fluorescence quantitative results of cellular uptake of different samples in pre‐stimulated EaRASMCs (I: MSCE, II: MMS/MSCE, III: MMS/MSCE/CAT). (Scale bars = 10 µm, *n* = 5). C) Cellular uptake efficiency of MSCE, MMS/MSCE, and MMS/MSCE/CAT in pre‐stimulated EaRASMCs (*n* = 5). D,E) Fluorescence images and corresponding normalized fluorescence quantitative results of expression of elastin (green) deposition in EaRASMCs cultures (I: Model, II: MSCE, III: MMS/MSCE, IV: MMS/MSCE/CAT). (Scale bars = 10 µm, *n* = 3). F,G) Fluorescence images and corresponding normalized fluorescence quantitative results of ROS levels in EaRASMCs. Cells in the normal groups were treated with medium alone, while the model groups were stimulated with TNF‐α and IL‐1β. For three treatment groups, cells were pretreated with different samples, followed by stimulation with TNF‐α and IL‐1β (I: Normal, II: Model, III: MSCE, IV: MMS/MSCE, V: MMS/MSCE/CAT). (Scale bars = 10 µm, *n* = 6). H) Normalized fluorescence quantification of ROS. Cells in the normal groups were treated with medium alone, while the model groups were stimulated with TNF‐α and IL‐1β. For three treatment groups, cells were pre‐stimulated with TNF‐α and IL‐1β, followed by treatment with different samples (I: Normal, II: Model, III: MSCE, IV: MMS/MSCE, V: MMS/MSCE/CAT).(*n* = 6). I, K, and M) The protein levels of MMP2, TIMP2, and LOX in pre‐stimulated EaRASMCs followed by different formulations treatment (I: Model, II: MSCE, III: MMS/MSCE, IV: MMS/MSCE/CAT). J, L, and N) WB analysis of β‐actin normalized expression of MMP2, TIMP2 and LOX proteins shown relative to values in model group (*n* = 3). Experimental data are mean ± SD of samples in a representative experiment. B, C, E, G, and H) *P < 0.05, **P < 0.01, and ***P < 0.001, determined by one‐way ANOVA. J, L, and N) ∗ denotes p < 0.05 compared to the model group, # denotes p < 0.05 compared to MSCE treated samples, $ denotes p < 0.05 compared to MMS/MSCE treated sample, determined by one‐way ANOVA.

Nanomotors are expected to exert their biological activities on AAA‐phenotyped EaRASMCs. We first verified the effect of different nanoparticles on the cellular activity of EaRASMCs and found that cellular viability remained consistently above 90% after incubation with various concentration of different samples for 24 h (Figure S8, Supporting Information). A concentration of 200 µg mL^−1^ was identified as the therapeutic dose. The subsequent evaluation of cellular uptake in EaRASMCs involved staining MSCE with Cy5 dye to trace MSCE (1.04 µg mL^−1^), MMS/MSCE (200 µg mL^−1^), and MMS/MSCE/CAT (200 µg mL^−1^), with equivalent exosome concentrations used to ensure uniform fluorescence intensity across all samples. Fluorescence imaging revealed that the red fluorescence intensity in the MMS/MSCE/CAT‐treated group peaked, whereas that in the MMS/MSCE‐treated group was similar to that of the MSCE‐treated group (**Figure** **4**A; Figure S9, Supporting Information). The quantitative results further demonstrated that the fluorescence intensity in the MMS/MSCE/CAT‐treated group was 3.5‐fold that of the MSCE‐treated group (Figure 4B). Calculations of cellular uptake rates across different samples validated these findings, with the MMS/MSCE/CAT‐treated group exhibiting the highest uptake rate at ≈23.1% (that is, 3.5‐fold that of the MSCE‐treated group) (Figure 4C). These results highlight the efficient endocytosis of MMS/MSCE/CAT by EaRASMCs. To evaluate the effects of CAT within MMS/MSCE/CAT on the increased cellular uptake, MMS was labeled with Cy5 dye to trace MMS/CAT, MMS/MSCE, and MMS/MSCE/CAT at a sample concentration of 200 µg mL^−1^. In pre‐stimulated EaRASMCs, higher fluorescence intensities were observed in the MMS/CAT‐and MMS/MSCE/CAT‐treated groups, which were 3.8‐ and 4.0‐fold that of the MMS/MSCE‐treated group, respectively (Figure S10, Supporting Information). These results indicate that the enhanced cellular uptake rate may be attributed to the enzyme‐driven effect of CAT.

After confirming that the nanomotors were efficiently internalized by EaRASMCs, we assessed their biological effect on these cells. Elastic matrix regenerative repair is expected to mitigate or reverse the progression of AAA. Elastin protein in EaRASMCs treated with various samples were subjected to immunofluorescence staining to investigate the effect of MMS/MSCE/CAT on the promotion of elastic deposition. The green fluorescence, indicative of elastin, was more pronounced in MMS/MSCE/CAT‐treated EaRASMCs compared to that in the model group (Figure 4D), which was 1.6‐fold higher than that in the MSCE‐treated group. Although the MSCE and MMS/MSCE groups exhibited enhanced fluorescence, quantitative analyses revealed no significant differences between these groups and the model group (Figure 4E). These findings highlight the substantial enhancement of elastin deposition by MMS/MSCE/CAT in EaRASMCs. Desmosine is an unusual, tetrafunctional, pyridinium ring‐containing amino acid, unique to mature cross‐linked elastins.^[^ ^27^ ^]^ To further assess whether the deposited elastin was cross‐linked, we conducted an enzyme linked immunosorbent assay (ELISA) to measure desmosine content in cell layer cultures treated with various samples. We observed a significant increase in desmosine content following MMS/MSCE/CAT treatment, which correlates with the trend observed in elastin fluorescence quantification (Figure S11, Supporting Information). These findings suggest that the elastin deposited in the cell cultures is mature elastin.

Local oxidative stress contributes to the progression and exacerbation of AAA. Therefore, we evaluated the in vitro antioxidant capabilities of various samples. EaRASMCs stimulated with inflammatory cytokines (TNF‐α and IL‐1β) exhibited significantly elevated ROS levels. Pretreatment with MSCE, MMS/MSCE, or MMS/MSCE/CAT mitigated ROS levels in EaRASMCs. In particular, the MMS/MSCE/CAT‐treated group exhibited a notable reduction in green fluorescence, indicating reduced ROS production in AAA‐phenotype EaRASMCs. Furthermore, both MSCE and MMS/MSCE treatments resulted in reduced ROS levels compared to those in the model group (Figure 4F,G), possibly because of inherent exosomal therapeutic properties. MSCE intervention in VSMCs post‐inflammatory stimulation resulted in reduced transforming growth factor (TGF)‐β1 expression.^[^ ^8^ ^]^—a known inducer of ROS production in VSMCs.^[^ ^28^ ^]^ This modulation of TGF‐β signaling by exosomes may explain the observed reduction in ROS levels. Moreover, we evaluated the antioxidant effects of different samples on the cellular milieu of EARASMCs under ROS activation. After stimulation with inflammatory factors, EaRASMCs were subjected to various treatments. Notably, treatment with MMS/MSCE/CAT effectively reduced the ROS levels, with no significant ROS reduction observed in the MSCE‐ and MMS/MSCE‐treated groups (Figure 4H). It was observed that MSCE, MMS/MSCE, and MMS/MSCE/CAT inhibited ROS generation in an inflammatory setting, whereas only MMS/MSCE/CAT demonstrated efficacy in scavenging ROS. This observation indicates a possible role for MSCE and CAT in inhibiting ROS generation in the inflammatory environment, with CAT also being responsible for ROS scavenging.

The overexpression of matrix metalloproteinases (MMPs) in AAA tissues contributes to ECM degradation, thereby exacerbating AAA progression. Consequently, mitigating MMPs expression is a promising strategy for alleviating AAA progression. We used inflammatory factor stimulation to induce MMP2 overexpression in EaRASMCs. Treatment with MMS/MSCE/CAT resulted in the most significant downregulation of zymogen MMP2, active MMP2, and total MMP2 expression levels compared to alternative interventions (Figure 4I,J). This indicates a strong inhibitory effect of MMS/MSCE/CAT on MMP2 expression and activity. Moreover, the regulation of MMP activity depends on the balance of tissue inhibitors of metalloproteinases (TIMPs), and the MMP/TIMP ratio affects the extent of ECM protein degradation and tissue remodeling.^[^ ^29^ ^]^ WB analysis revealed a significant upregulation of TIMP2 protein expression following MMS/MSCE/CAT treatment, with levels 2.3‐fold higher than those observed in the MSCE‐treated group (Figure 4K,L). Collectively, these findings indicate that MMS/MSCE/CAT treatment promotes an anti‐protein hydrolysis milieu in the EaRASMC culture model by suppressing MMP2 expression and enhancing the expression of its natural tissue inhibitor. Furthermore, we explored the expression of lysyl oxidase (LOX), an enzyme pivotal to ECM formation and repair, through elastin and collagen crosslinking.^[^ ^30^ ^]^ MMS/MSCE/CAT treatment significantly upregulated LOX protein expression by 2.6‐fold compared to MSCE treatment (Figure 4M,N), indicating that nanomotors play a beneficial role in enhancing the cross‐link stabilization of the fibroelastic matrix within aneurysmal SMCs.

In addition to VSMCs, inflammatory cells are prevalent in AAA. Inflammatory macrophages, which accumulate extensively in the vessel wall, overexpress pro‐inflammatory mediators such as TNF‐α and IL‐1β. These mediators, in turn, regulate the recruitment and activation of additional immune cells and contribute to VSMC apoptosis.^[^ ^31^ ^]^ To investigate the anti‐inflammatory effects of nanomotors, we assessed their impact on macrophages. RAW264.7 cells were pretreated with 100 ng mL^−1^ lipopolysaccharides for 24 h and subsequently exposed to various treatments. ELISA analysis of the cell supernatants revealed that TNF‐α and IL‐1β levels were significantly reduced in samples treated with MMS/MSCE/CAT. Notably, TNF‐α levels approached those of the normal control group, indicating that MMS/MSCE/CAT effectively exerts an anti‐inflammatory effect in macrophages (Figure S12, Supporting Information).

### 2.4 PI3K/Akt Signaling Pathway Activated in MMS/MSCE/CAT‐Treated EaRASMCs

**Figure 5:**
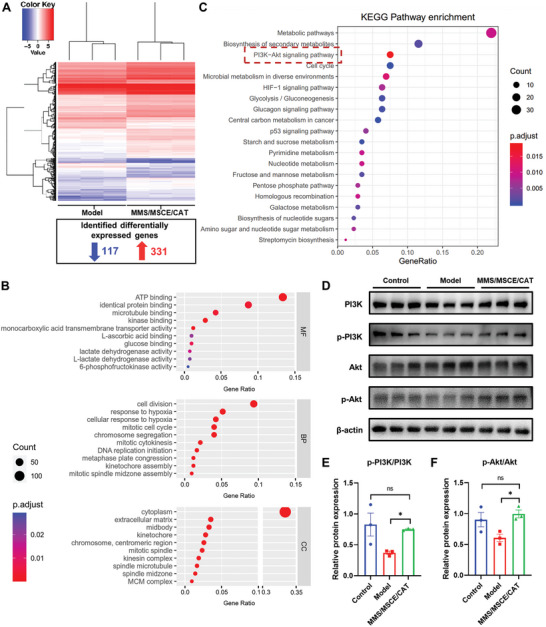
PI3K/Akt signaling pathway activated in MMS/MSCE/CAT‐treated EaRASMCs. A) Clustering of differentially expressed genes (DEGs), red indicates genes with high expression, blue indicates genes with low expression. B) GO functional analysis of DEGs. The size of circles indicated the numbers of DEGs. The red bar indicates a small padj value and the blue bar indicates a large padj value. C) KEGG pathways enrichment of DEGs. KEGG enrichment bubble diagram showed the top 20 KEGG pathways. The size of circles indicated the numbers of DEGs. The red bar indicates a large padj value and the blue bar indicates a small padj value. D) WB validation of the PI3K/Akt signaling pathway. Protein expression of PI3K, p‐PI3K, Akt, p‐Akt, and E) relative protein expression of p‐PI3K/PI3K and F) p‐Akt/Akt. Experimental data are means ± SD of samples in a representative experiment (*n* = 3). *P < 0.05, **P < 0.01, and ***P < 0.001, determined by one‐way ANOVA.

To further investigate the molecular mechanisms underlying the effects of MMS/MSCE/CAT on aneurysmal SMCs, we performed transcriptome sequencing to elucidate differential gene expression profiles. Our analysis revealed 448 differentially expressed genes (DEGs) between the AAA model group and the nanomotor‐treated group. Of these, 331 genes were significantly upregulated, while 117 genes were significantly downregulated (**Figure** **5**A; Figure S13, Supporting Information). Notably, genes encoding αSMA, SM22α, and CNN1 (Acta2, Tagln, and Cnn1) exhibited significant upregulation, which is indicative of possible phenotypic switching in SMCs induced by MMS/MSCE/CAT treatment. Moreover, the upregulation of certain ECM genes (e.g., Col12a1 and Col3a1) suggests their potential roles in ECM structure maintenance and repair. Additionally, the decreased expression of protease genes (e.g., MMP1/10/12/13) implies a possible protective effect against MMP‐mediated ECM damage.

Furthermore, gene ontology functional enrichment analysis categorized the DEGs into molecular function (MF), biological process (BP), and cellular component (CC). The top ten significant entries for each module are shown in Figure 5B. This visualization displays the gene ratio along the horizontal axis, the name of the enriched entries on the vertical axis, and the size of each scatter point representing the number of targets involved. Entries of high importance are highlighted in red. The molecular functions are mainly related to ATP binding, identical protein binding, microtubule binding, kinase binding, and other molecular functions. Biological processes are mainly associated with cell division and responses to hypoxia. The cellular components are mainly related to the cytoplasm, ECM, and other cellular components.

The DEGs were further analyzed for KEGG enrichment, which revealed the top 20 KEGG pathways depicted in Figure 5C. These results suggest that MMS/MSCE/CAT may exert its effects through multiple pathways, including metabolic pathways, biosynthesis of secondary metabolites, PI3K/Akt signaling pathway, HIF‐1 signaling pathway, and p53 signaling pathway. The node size and color in the bubble graph correspond to the number of associated genes and padj value, respectively. Larger nodes represent more target genes, and colors shifting from purple to red indicate increasing padj values. Previous research has elucidated that PI3K/Akt pathway activation upregulates proelastin expression,^[^ ^32^ ^]^ while the lack of PI3Kα downregulates elastin and protofibrillar protein expression in SMCs.^[^ ^33^ ^]^ This highlights the role of PI3K in elastic fiber homeostasis. Furthermore, a complex positive feedback loop involving HIF‐1, PI3K/Akt, and LOX has been identified. In this loop, HIF‐1 activation induces LOX expression, and LOX activity subsequently activates the PI3K/Akt pathway, amplifying HIF‐1α expression at the translational level.^[^ ^34^ ^]^ These findings are consistent with our sequencing results, suggesting the potential impact of PI3K/Akt and HIF‐1 signaling pathway activation on elastic matrix regenerative repair. Additionally, PI3K/Akt signaling predominantly enhances proliferation and suppresses apoptosis of VSMCs.^[^ ^35^ ^]^ Akt2 deficiency in the aorta leads to a significant increase in apoptosis among VSMCs,^[^ ^36^ ^]^ whereas Akt activation proliferates and hinders apoptosis in aortic wall VSMCs, offering protection against aneurysm dilation.^[^ ^37^ ^]^ WB analysis revealed increased levels of both p‐PI3K/PI3K and p‐Akt/Akt after MMS/MSCE/CAT treatment (Figure 5D–F), confirming the activation of the PI3K/Akt pathway.

### 2.5 In Vivo Targeting Capability of the Nanomotors

**Figure 6:**
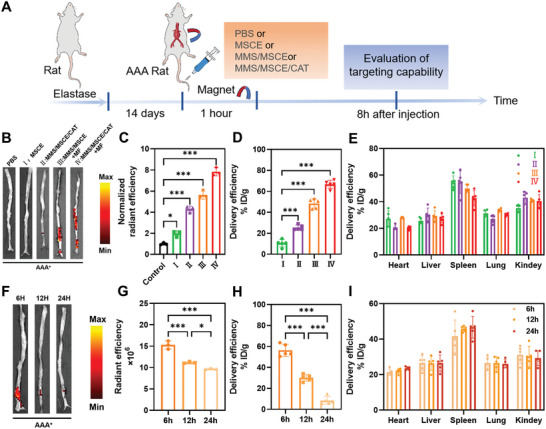
Targeting capability of intravenously delivered different samples in AAA rats in vivo. A) Schematic illustration of regimens for in vivo targeting studies. B) Representative ex vivo fluorescence images and C) quantification results showing the accumulation of Cy5‐labeled samples in aneurysmal aortas (*n* = 3). Biodistribution of different samples in D) aneurysmal aortas and E) other organs (I: MSCE, II: MMS/MSCE/CAT, III: MMS/MSCE+MF, IV: MMS/MSCE/CAT+MF). (*n* = 5). F) Ex vivo fluorescence images and G) quantification results indicate the retention effect of accumulated Cy5‐labeled MMS/MSCE/CAT in aneurysmal lesions (*n* = 3). Biodistribution of MMS/MSCE/CAT in H) aneurysmal aortas and I) other organs after intravenous injection for different times. MSCE was labeled with Cy5 dye (*n* = 5). Experimental data are means ± SD of samples in a representative experiment. *P < 0.05, **P < 0.01, and ***P < 0.001, determined by one‐way ANOVA.

In vitro experiments underscore the potential of nanomotors to effectively target AAA via chemotaxis toward high H_2_O_2_ concentrations produced by EaRASMCs, demonstrating significant biological effects. We further evaluated the ability of nanomotors to target AAA under the combined effect of magnetotropism and chemotaxis in a rat AAA model. MSCE and MMS/MSCE/CAT were administered intravenously, and the other two groups additionally applied MF to the aneurysmal region for 1 h after the injection of MMS/MSCE or MMS/MSCE/CAT. The rats were euthanized at 8 h post‐administration, and isolated aortas were imaged in vitro to validate the active targeting efficacy of the therapeutic agents against AAA (**Figure** **6**A).

It was found that upon injecting MMS/MSCE/CAT and adding MF (dual‐driven), the red fluorescent area in the isolated aorta reached its maximum extent (Figure 6B). Subsequent quantification of fluorescence confirmed this observation, with the fluorescence intensity in the aorta of group IV being 4.0, 1.8, and 1.4 times higher than that in groups I (MSCE), II (chemotactic nanomotor), and III (magnetically responsive nanomotor), respectively (Figure 6C). This highlights the enhanced targeting capability of nanomotors achieved through dual functionalization. To comprehensively analyze the targeting efficacy across various organs, we isolated and quantified tissues from rats 8 h post‐tail vein injection. Tissue samples were ground, lysed, and subjected to fluorescence intensity measurements to quantitatively analyze sample distribution. Notably, groups III and IV exhibited significantly heightened AAA abdominal aortic accumulation of 48.4% ID/g and 66.7% ID/g, respectively, compared to groups I and II (Figure 6D). Moreover, the intraorgan distribution in groups III and IV was diminished relative to that in groups I and II (Figure 6E). Increased aortic fluorescence accumulation after MMS/MSCE/CAT injection compared to direct MSCE injection was observed. However, the aortic fluorescence accumulation was reduced compared to MMS/MSCE injection, followed by MF navigation. This observation suggests that magnetically guided delivery may be more helpful in overcoming resistance to blood flow and for precise localization at the disease site in a complex arterial flow environment. In comparison to the above methodologies, the injection of MMS/MSCE/CAT followed by additional MF navigation achieved maximal fluorescence accumulation. This phenomenon likely stems from the nanomotor enrichment around diseased tissues induced by the applied MF, with additional augmentation via chemotaxis. This combination enhances their anchoring capabilities for optimal targeting efficacy.

To investigate the dynamic evolution of MMS/MSCE/CAT distribution in vivo, the rats were euthanized at 6, 12, or 24 h post‐treatment. The isolated aortas were then subjected to in vitro imaging, and different organ tissues were segregated for quantitative analysis. At 6 h post‐injection, the abdominal aorta exhibited the largest red fluorescence area attributable to MMS/MSCE/CAT (Figure 6F), with subsequent quantitative analysis revealing a gradual decline in fluorescence intensity over time (Figure 6G). Biodistribution results further revealed that MMS/MSCE/CAT peaked in the AAA wall at 6 h post‐injection, with a concentration of 56.3% ID/g, followed by a gradual decrease in fluorescence intensity over time (Figure 6H). MMS/MSCE/CAT accumulates in multiple organs, with predominant accumulation in the spleen (Figure 6I). In addition, the biodistribution of MMS/MSCE/CAT was also detected by Cy5 fluorescent labeling of MMS. A similar pattern of fluorescence accumulation was observed in abdominal aorta and other major organs (Figure S14, Supporting Information).

### 2.6 In Vivo Protection Effect of the Nanomotors on AAA

**Figure 7:**
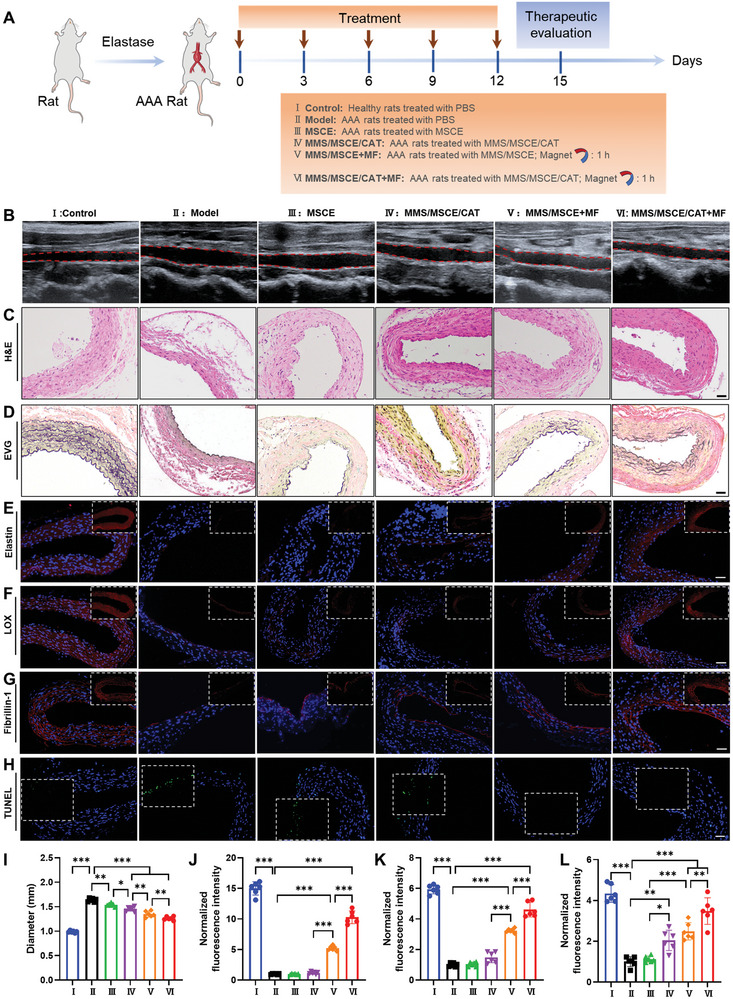
Therapeutic effects of different samples in AAA rats in vivo. A) Schematic illustration of establishment of elastase‐induced AAA in rats and treatment regimens for different groups (I: Control, II: Model, III: MSCE, IV: MMS/MSCE/CAT, V: MMS/MSCE+MF, VI: MMS/MSCE/CAT+MF). B) Representative ultrasound images of the longitudinal sections of abdominal aortas for different groups. C) H&E‐stained histopathologic section of the aorta indicating the morphology of the aneurysmal aorta. D) EVG‐stained histopathologic section of the aorta indicating the elastin degradation in aneurysmal aortas. E) Immunofluorescence images showing the expression of elastin (red) in the abdominal aorta. F) Immunofluorescence images showing the expression of LOX (red) in the abdominal aorta. G) Immunofluorescence images showing the expression of Fibrillin‐1 (red) in the abdominal aorta. H) Microscopy images of TUNEL‐stained sections of aortic tissues from rats subjected to different treatments. I) The mean maximal diameter of the abdominal aortas quantified by micro‐ultrasound. The quantitative analysis of the expression of J) elastin, K) LOX, and L) Fibrillin‐1 in aneurysm after different treatments. Scale bars = 50 µm. Experimental data are means ± SD of samples in a representative experiment (*n* = 6). *p < 0.05, **p < 0.01, ***p < 0.001, determined by one‐way ANOVA.

The effects of various treatments on elastase‐induced AAA in rats were examined (**Figure** **7**A). As previously described, MSCE and MMS/MSCE/CAT were administered intravenously, with the additional application of MF to the aneurysmal region for 1 h following the injection of MMS/MSCE or MMS/MSCE/CAT in the other two groups. Longitudinal and cross‐sectional microscopic ultrasound imaging was conducted 14 days postoperatively and 15 days post‐treatment, respectively. The images taken 14 days postoperatively depicted a mean abdominal aorta diameter of approximately 0.96 mm under normal physiological conditions. In contrast, the abdominal aorta of the model group exhibited significant dilation, with a mean diameter of ≈1.50 mm (Figure S15, Supporting Information). This confirmed the successful AAA model construction. Treatment results showed that aneurysm expansion was slower in groups III and IV than in the PBS‐treated group, and the aneurysm diameter was reduced in groups V and VI compared with the pretreatment AAA diameter, indicating the reversal of aneurysm progression (Figure 7B). Statistical analysis showed that the mean arterial diameter of groups V and VI after treatment was ≈1.35 and 1.26 mm, which decreased to 90.0% and 84.0% of the pretreatment vascular diameter, and ≈82.3% and 76.8% of the diameter of the PBS‐treated group (Figure 7I). These results prove that the nanomotors reversed AAA progression.

Further histological examination of the aneurysmal aortic sections after staining with H&E or EVG revealed significant damage to the abdominal aorta of the model group, characterized by thinning of the media layer and severe elastin fiber rupture. In contrast, these pathological abnormalities were ameliorated in groups V and VI, which exhibited substantial thickening of the media layer and elastic matrix regeneration (Figure 7C,D). Immunofluorescence staining of elastin in vascular tissues revealed minimal fluorescence in the model group, and increased fluorescence expressions in groups V and VI compared to those in groups III and IV. Notably, group VI exhibited the most pronounced expression (Figure 7E; Figure S16, Supporting Information). Quantification of elastin fluorescence was achieved with the fluorescence levels of the PBS‐treated group serving as the normalization standard. The highest fluorescence expression was observed in group VI, which was 10.2, 8.6, and 2.0 times higher than those in groups III, IV, and V, respectively (Figure 7J). In addition to elastin, LOX expression in vascular tissues was assessed using immunofluorescence staining across all groups. LOX fluorescence was notably elevated in groups V and VI compared to that in groups III and IV, with group VI displaying the most pronounced expression (Figure 7F; Figure S17, Supporting Information). Quantification confirmed that the highest fluorescence intensity was observed in group VI, which was 4.5, 3.1, and 1.4 times higher than those in groups III, IV, and V, respectively (Figure 7K). Given that fibrillin is integral to microfibrils in the elastic extracellular matrix and play a crucial role in elastin assembly into elastic fibers, we conducted immunofluorescence staining for fibrillin‐1. The results demonstrated that group VI exhibited the highest fluorescence intensity (Figure 7G; Figure S18, Supporting Information). Quantification revealed that the fluorescence intensity in group VI was 3.1, 1.7, and 1.3 times greater than those in groups III, IV, and V, respectively (Figure 7L). Desmosine can be used as an indicator to predict the disease severity and clinical prognosis of AAA patients.^[^ ^38^ ^]^ We measured serum desmosine levels in rats across different treatment groups and found that group VI exhibited the most significant reduction in serum desmosine levels compared to the model group (Figure S19, Supporting Information). This result aligns with previously reported clinical patient data, which indicate that larger AAA diameters are associated with higher serum desmosine levels.^[^ ^38^ ^]^ These results suggest that the nanomotor not only facilitates elastic matrix regeneration but may also enhance elastic matrix cross‐linking.

Activation of the PI3K/Akt signaling pathway in EaRASMCs through MMS/MSCE/CAT treatment is closely related to VSMCs apoptosis. Apoptosis in AAA was evaluated by TUNEL staining of vascular tissues. Significantly reduced cell apoptosis was observed in groups V and VI compared to the PBS‐treated group, whereas no significant improvement was noted in groups III and IV (Figure 7H; Figure S20, Supporting Information).

Biosafety and feasibility of nanomotors are essential for their application in the biomedical fields.^[^ ^39^ ^]^ To assess the biosafety of the prepared nanomotors, the rats injected with various samples for 24 h underwent routine blood analyses. Compared with rats injected with PBS via the tail vein, those treated with MSCE, MMS/MSCE, and MMS/MSCE/CAT did not show signs of anemia, thrombocytopenia, or abnormal white blood cell counts (Figure S21, Supporting Information). Additionally, at the end of the treatment, histopathological examination of the major organs (heart, liver, spleen, lungs, and kidneys) by H&E staining revealed no significant inflammatory infiltrates or histological abnormalities (Figure S22, Supporting Information).

### 2.7 MRI Capability of the Nanomotors on AAA

**Figure 8:**
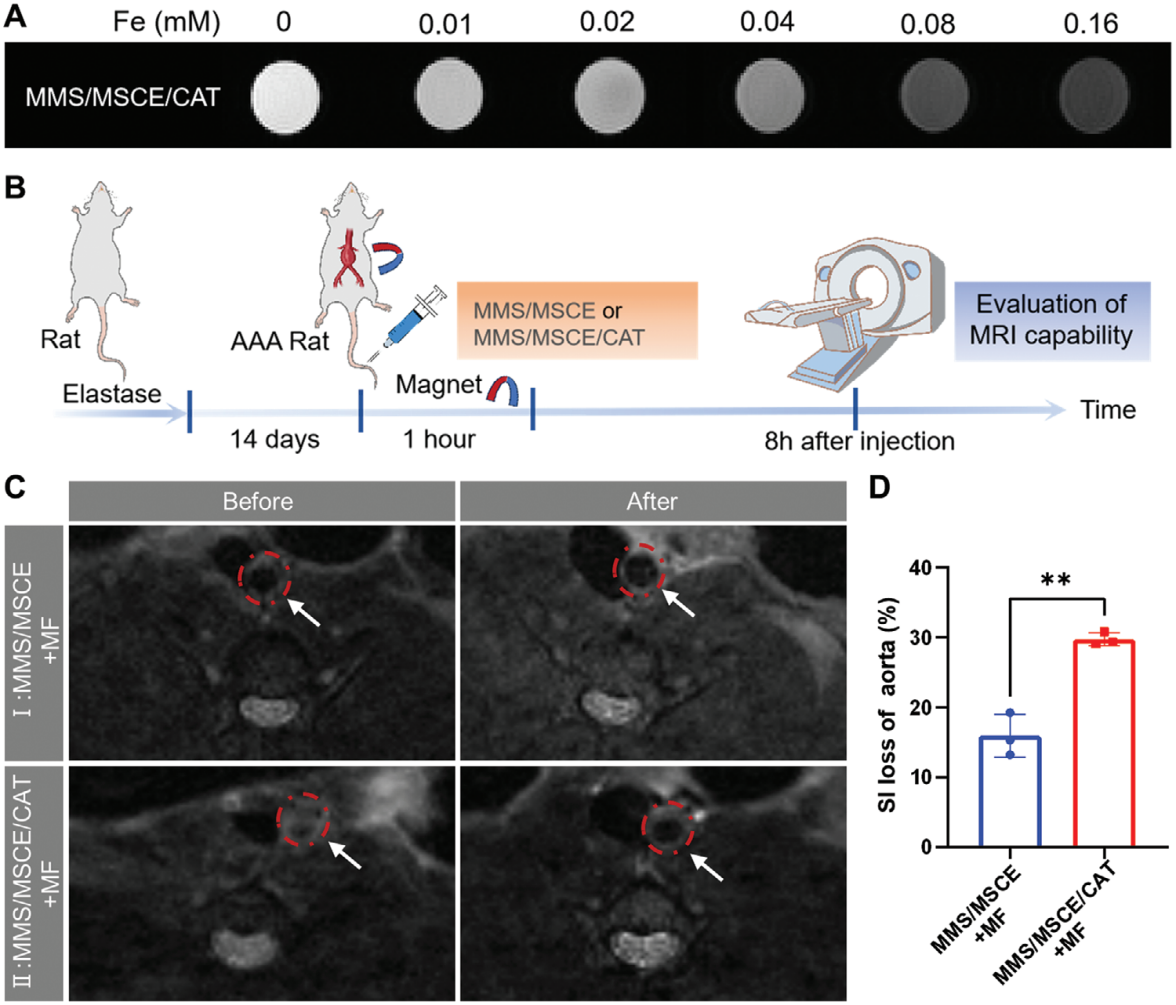
Magnetic resonance imaging (MRI) capability of nanomotors. A) In vitro MRI T_2_ imaging with different concentrations of MMS/MSCE/CAT. B) Schematic illustration of regimens for in vivo MRI studies. C) Serial in vivo MRI of AAA before and after the treatment of nanomotors. Red circles indicating the aneurysmal site. D) Quantitative analysis of the normalized signal intensity (%SI loss). Experimental data are means ± SD of samples in a representative experiment (*n* = 3). *P < 0.05, **P < 0.01, and ***P < 0.001, determined by unpaired Student's t test.

To evaluate the MRI capabilities of the nanomotors, in vitro MRI T_2_ imaging was initially performed on MMS/MSCE/CAT, which demonstrated a concentration‐dependent T_2_ imaging capability (**Figure** **8**A). The two groups with the best in vivo targeting performance were validated using in vitro MRI T_2_ imaging (Figure 8B). Ultrasound assessments confirmed that the arterial diameters of all rats with AAA were ≈1.5 mm prior to drug administration. MRI performed before treatment confirmed similar aneurysm areas with the largest diameters across the groups. After intravenous injection of MMS/MSCE or MMS/MSCE/CAT, MF was applied to the aneurysm area for 1 h, and in vivo MRI was performed 8 h later. Both groups exhibited significant T_2_ signal loss, with the MMS/MSCE/CAT group showing a more pronounced loss (Figure 8C). Quantitative analysis demonstrated that the group using MMS/MSCE/CAT experienced significantly greater %SI loss than the group using MMS/MSCE (29.8 ± 0.9% versus 15.9 ± 3.1%, p = 0.0017) (Figure 8D).

## 3 Conclusion

Recent studies have demonstrated that several treatments have proven effective in slowing AAA progression.^[^ ^40^ ^]^ However, reversing the progression of small aneurysms remains challenging. This requires promoting the regeneration of elastic fibers in the vessel walls to restore the mechanical and biochemical functions of the vasculature.^[^ ^41^ ^]^ Elastogenesis is vital for this process and mainly occurs during the fetal and neonatal stages. In AAAs, degraded or apoptotic VSMCs lack proper elastogenesis, hindering fiber repair.^[^ ^42^ ^]^ For structural restoration of the aortic wall, VSMCs require external stimuli to promote elastin regeneration and resist proteohydrolysis.

Exosomes derived from BM‐MSCs have the potential to repair the elastic matrix of aneurysmal SMCs.^[^ ^8^ ^]^ However, their efficacy in animal models remains unclear. Moreover, exosome therapy faces challenges such as insufficient targeting, and rapid clearance.^[^ ^10^, ^43^ ^]^ The high‐speed and complex blood flow environment in the arterial system further complicates the application of exosome therapy for cardiovascular diseases.^[^ ^11^ ^]^ To address the challenges of targeted delivery of exosomes mentioned above, we designed a dual‐drive nanomotor, MMS/MSCE/CAT, combining magneto‐ and enzyme‐driven capabilities. Upon injection into the tail vein, the nanomotors were guided to the low‐flow‐rate region near the AAA wall using an MF. MMS/MSCE/CAT responds to the high H_2_O_2_ environment of the AAA wall, exhibiting chemotactic behavior that allows for secondary targeting of the AAA and enhances the specificity of the therapy. The graded targeting strategy significantly enhanced the accumulation of MSCE at the aneurysm site in AAA rats, exhibiting notable efficacy. In the elastase‐induced AAA model, treatment with MMS/MSCE/CAT substantially reduced the AAA diameter compared to the pretreatment levels, effectively inhibiting disease progression. Mechanistically, MMS/MSCE/CAT enhanced the endocytosis efficiency of VSMCs. This facilitated elastic matrix regeneration and crosslinking, thereby promoting the restoration of the elastic fiber network. Moreover, the nanomotor reduced the ROS levels, MMPs activity, and apoptosis, collectively inhibiting AAA progression through multifaceted pathways. Transcriptome sequencing revealed potential associations between the therapeutic effects of MMS/MSCE/CAT and the activation of the PI3K/Akt signaling pathway.

Previous nanotherapies for the treatment of AAA, summarized in Table S1 (Supporting Information), primarily targeted mitigating disease‐contributing factors such as MMPs activity inhibition, inflammation reduction, ROS scavenging, and calcification reduction.^[^ ^44^ ^]^ While these strategies partially impede AAA progression, they fail to substantially affect arterial tissue repair, thus failing to reverse AAA advancement or restore arterial health. Recent studies have shown that pentagalloyl glucose (PGG)‐loaded nanoparticles conjugated with elastin antibody can restore the elastic lamina and reverse the aortic dilation,^[^ ^45^ ^]^ underscoring the critical role of promoting elastic matrix regeneration in AAA reversal. However, this study did not address the targeted delivery efficiency of the drug, and the accumulation of PGG in other organs could potentially lead to systemic effects. Therefore, enhancing the AAA‐targeting efficiency of existing nanotherapies and minimizing nonspecific nanomedicine distribution in major organs remains pivotal for advancing AAA treatment. In contrast, we introduced a novel approach by utilizing nanomotor technology to promote elastic matrix regeneration in an animal model of AAA. Although this approach did not fully restore the aneurysm to its normal state, it demonstrated a therapeutic potential for reversing AAA progression. Furthermore, the synergistic dual‐driven performance of magnetic guidance and chemical propulsion facilitated the enrichment and secondary targeting of nanoplatforms within AAA, significantly enhancing their targeting efficiency while mitigating nonspecific organ distribution. This innovative MMS/MSCE/CAT nanomotor presents a novel and promising therapeutic avenue not only for small AAA interventions but also for addressing other cardiovascular disease processes, offering a versatile platform for intervention.

Despite these advances, certain limitations of this study warrant further consideration. First, the molecular mechanism underlying the therapeutic efficacy of the nanomotors was elucidated primarily through transcriptome sequencing and related pathway analyses at the cellular level. This necessitates validation through animal sample sequencing to bolster the experimental findings. Second, although an elastase‐induced AAA rat model was used to validate nanomotor efficacy, its limitations in fully recapitulating AAA pathogenesis underscore the need to incorporate models with alternative pathological mechanisms to affirm the reliability of nanomotor efficacy. Finally, the mechanism of action of MSCE in AAA has not been exhaustively explored. Beyond its role in promoting elastic matrix regenerative repair, MSCE may exert additional effects, warranting further investigation. For instance, both experimental MSCE and MMS/MSCE treatments, even without CAT loading, demonstrated intracellular oxidative stress attenuation, possibly due to inherent exosomal therapeutic properties. Notably, MSCE intervention in VSMCs post‐inflammatory stimulation reduced TGF‐β1 expression,^[^ ^8^ ^]^ —a known inducer of ROS production in VSMCs.^[^ ^28^ ^]^ This modulation of TGF‐β signaling by exosomes might explain the reduced ROS levels, necessitating validation through further experimentation. In addition, studies have indicated that MSCE is linked to VSMCs calcification and can inhibit this process.^[^ ^46^ ^]^

## 4 Experimental Section

Methods and any associated references are available in the Supporting Information.

## Conflict of Interest

The authors declare no conflict of interest.

## Supporting information



Supporting Information

Supplemental Movie 1

Supplemental Movie 2

Supplemental Movie 3

Supplemental Movie 4

## Data Availability

The data that support the findings of this study are available from the corresponding author upon reasonable request.
