# Supplementary material for: Enhancing Exosomal Delivery to Abdominal Aortic Aneurysms using Magnetically Responsive Chemotactic Nanomotors for Elastic Matrix Regenerative Repair
Source: Adv Sci (Weinh). 2024 Oct 21;11(46):2405085. doi: 10.1002/advs.202405085 (PMC11633499; doi:10.1002/advs.202405085)
Supplement: Supplementary file 1 — Supporting Information [file ADVS-11-2405085-s005.docx]

**Supporting Information of**

**Enhancing Exosomal Delivery to Abdominal Aortic Aneurysms Using Magnetically Responsive Chemotactic Nanomotors for Elastic Matrix Regenerative Repair**

*Lulu Wang,^‡, [a]^ Yao Zhang,^‡, [b]^ Chun Mao,*^[a,b]^ and Xiaoqiang Li,*^[a]^*

‡These authors contributed equally.

[a] L. Wang, C. Mao, X. Li
Department of Vascular Surgery
Nanjing Drum Tower Hospital, Affiliated Hospital of Medical School, Nanjing University
Nanjing 210008, Jiangsu Province, China.
E-mail: flytsg@126.com; maochun@njnu.edu.cn

[b] Y. Zhang, C. Mao
National and Local Joint Engineering Research Center of Biomedical Functional Materials, School of Chemistry and Materials Science
Nanjing Normal University
Nanjing 210023, Jiangsu Province, China.

**Table of Contents**

**Experimental section**

**Figure S1** (A)TEM image of MSCE, (B) NTA analysis of MSCE, (C) WB analysis of exosome marker proteins in MSCE and MMS/MSCE/CAT.

**Figure S2** Standard curve of catalase activity at room temperature and catalase activity of different nanoparticles (*n* = 3). Triangles indicate MMS/MSCE. Squares indicate MMS/MSCE/CAT.

**Figure S3** Magnetic separation experiment.

**Figure S4** Validation of primary arterial smooth muscle cells (Red fluorescence: α-SMA; Blue fluorescence: nucleus; Scale bar, 100 μm).

**Figure S5** Cellular H_2_O_2_ levels after 10 ng mL^-1^ TNF-α and 10 ng mL^-1^ IL-1β stimulation of EaRASMCs for 0, 4, 8, 12, and 24 h. Data are mean ± SD (*n* = 6).

**Figure S6** Schematic illustration of dynamic microfluidic model with Cy5-labeled samples in buffer (II), prestimulated EaRASMCs lysate in buffer (III -2) and normal VSMCs lysate in buffer (I or III -1).

**Figure S7** Corresponding normalized fluorescence intensities of MMS/MSCE flow in the presence of (A) buffer I and buffer III-1, and (B) buffer I and buffer III-2 on two sides; Corresponding normalized fluorescence intensities of MMS/MSCE/CAT flow in the presence of (C) buffer I and buffer III-1, and (D) buffer I and buffer III-2 on two sides.

**Figure S8** Cell viability of EaRASMCs after incubation with various doses of different samples for 24 h. (A) MMS/MSCE; (B) MMS/CAT; (C) MMS/MSCE/CAT. Data are mean ± SD (*n* = 6).

**Figure S9** Fluorescence images showing cellular uptake of MSCE, MMS/MSCE and MMS/MSCE/CAT in EaRASMCs. MSCE was stained with Cy5 (red), nuclei were stained with DAPI (blue) and cell membranes were stained with DiO (green). Scale bar = 10 μm.

**Figure S10** (A) Representative fluorescence images of cellular uptake of MMS/MSCE, MMS/CAT and MMS/MSCE/CAT in pre-stimulated EaRASMCs (Scale bars = 10 μm) and (B) the corresponding normalized fluorescence quantification. Data are mean ± SD (*n* = 5). *P < 0.05, **P < 0.01, and ***P < 0.001, determined by one-way ANOVA.

**Figure S11** ELISA analysis of desmosine levels in EaRASMCs. Data are mean ± SD (*n* = 6). *P < 0.05, **P < 0.01, and ***P < 0.001, determined by one-way ANOVA.

**Figure S12** TNF-α and IL-1β levels in culture supernatants collected from RAW264.7 cells cultures. Cells in the normal groups were treated with medium alone, while the model groups were stimulated with 100 ng mL^-1^ LPS. For three treatment groups, cells were pre-stimulation with LPS, followed by treatment with different samples (I: Normal, II: Model, III: MSCE, IV: MMS/MSCE, V: MMS/MSCE/CAT). Data are mean ± SD (*n* = 6). *P < 0.05, **P < 0.01, and ***P < 0.001, determined by one-way ANOVA.

**Figure S13** Volcano plot of differentially expressed genes (Fold change ≥2.0 or ≤0.5, padj <0.05; Blue represents significantly down-regulated genes, yellow represents significantly up-regulated genes, and gray represents non-significantly different genes).

**Figure S14** Biodistribution of MMS/MSCE/CAT in (A) aneurysmal aortas and (B) other organs after intravenous injection for different times. MMS was labeled with Cy5 dye. Data are mean ± SD (*n* = 5). *P < 0.05, **P < 0.01, and ***P < 0.001, determined by one-way ANOVA.

**Figure S15** Representative ultrasound images of the longitudinal and transverse sections of abdominal aortas for different groups.

**Figure S16** Immunofluorescence images showing the expression of elastin (red) in the abdominal aorta. Scale bar = 50 μm; the corresponding enlarged views with a scale bar of 25 μm.

**Figure S17** Immunofluorescence images showing the expression of LOX (red) in the abdominal aorta. Scale bar = 50 μm; the corresponding enlarged views with a scale bar of 25 μm.

**Figure S18** Immunofluorescence images showing the expression of Fibrillin-1 (red) in the abdominal aorta. Scale bar = 50 μm; the corresponding enlarged views with a scale bar of 25 μm.

**Figure S19** ELISA analysis of serum desmosine levels in AAA rats subjected to different treatments. Data are mean ± SD (*n* = 6). *P < 0.05, **P < 0.01, and ***P < 0.001, determined by one-way ANOVA.

**Figure S20** Microscopy images of TUNEL-stained sections of aortic tissues from AAA rats subjected to different treatments. Scale bar = 50 μm; the corresponding enlarged views with a scale bar of 25 μm.

**Figure S21** Quantification of typical hematological parameters at 24 h after treatment with different samples in rats. The blood levels of (A) RBC, (B) WBC, (C) HGB and (D) PLT. Data are mean ± SD (*n* = 3).

**Figure S22** H&E-stained pathological sections of typical major organs excised from rats at day 15 after treatment with different samples. Scale bar, 100 µm.

**Movie S1** Motion behavior of MMS/MSCE in normal VSMCs environment for 10 s.

**Movie S2** Motion behavior of MMS/MSCE/CAT in normal VSMCs environment for 10 s.
**Movie S3** Motion behavior of MMS/MSCE in pre-stimulated EaRASMCs environment for 10 s.
**Movie S4** Motion behavior of MMS/MSCE/CAT in pre-stimulated EaRASMCs environment for 10 s.

**Table S1** Summary of previous nanotherapies for AAA treatment.

Experimental section

**Materials and chemicals**

(1,2-bis(triethoxysilyl)ethane (TESPTS) and catalase (CAT) were also purchased from Aladdin Chemistry Co., Ltd. Tetraethylorthosilicate (TEOS), hexadecyl trimethyl ammonium bromide (CTAB), hydrochloric acid (HCl), sodium hydroxide (NaOH) and ammonium hydroxide (NH_3_·H_2_O) were bought from Sinopharm Chemical Reagent Co., Ltd. 4-(N-Maleimidomethyl) cyclohexane-1-carboxylic acid 3-sulfo-N-hydroxysuccinimide ester sodium salt (sulfo-SMCC) and (3-Mercaptopropyl) trimethoxysilane (MPTES) were purchased from Aladdin Biochemical Technology Co., Ltd. (Shanghai, China). Superparamagnetic iron oxide nanoparticles (SPION) was purchased from Beiou Biotechnology Co., Ltd (Shaanxi, China). 3,3′-dioctadecyloxacarbocyanine perchlorate (DiO), Cyanine 5 (Cy5) and dimethyl sulfoxide (DMSO) were purchased from Shanghai Yuanye Biotech (Shanghai, China). Tumor necrosis factor (TNF)-α and interleukin-1β (IL1β) were purchased from PeproTech Inc. 4’,6-Diamidino-2-phenylindole dihydrochloride (DAPI) was obtained from Sigma Aldrich Co., Ltd. Bicinchonic acid (BCA) assay (Thermo Fisher Scientific), DMEM/F12 (1:1) (Hyclone), DMEM high glucose (Gibco), Phosphate buffer saline (PBS) (Gibco) and Pen-Strep (Gibco) were purchased from Shanghai Xinyu Biotechnology Co., Ltd. Cell counting kit-8 (CCK-8) was purchased from Beyotime Biotechnology (Shanghai, China). Fetal bovine serum (FBS) and exosome-free FBS was provided by Huaying Biomedical Technology Co., LTD (Shanghai, China).

**Isolation and Extraction of MSCE**

MSCE was prepared according to a typical protocol^[1]^. Briefly, BM-MSCs were cultured in DMEM/F12 (1:1) supplemented with 10% FBS, and 1% Pen-Strep (v/v). When the cells have grown to cover 70% of the culture flask, the upper culture medium was replaced with 10% v/v exosome-free FBS-supplemented medium. After 48 h of culture, the upper culture medium was collected and stored at -80°C until use. The culture medium was centrifuged at 300 g at 4°C for 10 min to remove dead cells and cell fragments and the upper culture medium was further centrifuged at 2000 g at 4°C for 10 min and at 10,000 g for 30 min. The supernatant was transferred into poly-carbonate tubes and centrifuged at 100,000 g for 70 min (Type 50.2 Ti rotor, Beckman Coulter, Fullerton, CA). The pellets were dispersed in PBS by gentle pipetting, and protein concentration was measured using BCA assay. If staining was required, add Cy5 (10 µL, 1 mg mL^-1^) or DiO (0.5 mL, 20 µM). The upper cell culture medium was discarded, and the lower MSCE was collected. The MSCE, DiO labeled MSCE and Cy5 labeled MSCE were dispersed in PBS and stored at -80°C, respectively.

**Synthesis of Bowl-shaped Mesoporous Silica Nanoparticles (MMS)**

20 mg SPION was added to a mixture of water (150 mL), ethanol (60 mL), CTAB (0.32 g), and NH_3_·H_2_O (2.0 mL, 25 - 28 wt%). It is then heated in a 35°C water bath and a mixture of TESPTS (0.2 mL) and TEOS (0.5 mL) is added dropwise with strong agitation. After agitation for 24 h, the product was collected by centrifugation (8000 rpm, 10 min) and washed three times with ethanol. The product was dispersed into NaOH (63 mL) in water (0.48 M), etched for 30 min at room temperature, and washed 3 times (4000 rpm, 10 min) with ultrapure water. The product was placed in a Soxhlet extractor, and a mixed solution of HCl (480 μL, 37%) and ethanol (240 mL) was added to a round-bottom flask and refluxed at 90°C for 12 h to extract and separate the CTAB template. The product is then washed three times (4000 rpm, 10 min) with ethanol centrifugation to collect the bowl-shaped MMS.

**Synthesis of Nanoparticles Coupled to MSCE (MMS/MSCE)**

The bowl-shaped MMS (20 mg) was dispersed in ethanol (20 mL), MPTES (20 μL) was added and placed on a shaker, the reaction was carried out at room temperature for 24 h, centrifugation (4000 rpm, 10 min), and the bowl of sulfhydryl modification (MMS-SH) was obtained by washing with ultrapure water for 3 times, and MMS-SH (2 mg) was dispersed in PBS buffer (2 mL), and then Sulfo-SMCC (2 mg) was placed on a shaker, and the reaction was carried out at room temperature for 24 h, and then centrifuged and washed with PBS buffer for 3 times (4000 rpm, 10 min) to obtain the product MMS-SMCC after reaction with sulfhydryl groups. If staining is required, add Cy5 fluorescent dye (10 μL, 1 mg mL^-1^). The obtained MMS-SMCCs were dispersed in PBS buffer (2 mL), then exosome dilution (2 μg μL^-1^, 20 μL) was added, and the reaction was placed on a shaker for 24 h at 4°C, followed by 3 centrifugation washes (4000 rpm, 10 min) with PBS buffer to obtain MMS/MSCE or Cy5-labeled MMS/MSCE. If the exosomes added are DiO-labeled MSCE, Cy5 and DiO dual-labeled MMS/MSCE (2 mg sample based on the quality of MMS is the standard).

**Synthesis of Nanomotors Loaded with Catalase (MMS/MSCE/CAT)**

The above products were dispersed to 2 mL with PBS buffer, CAT (2 mg) was added, and the unloaded CAT was removed by centrifugal washing with PBS buffer for 3 times (4000 rpm, 10 min) after 24 h of shaking reaction, and MMS/MSCE/CAT could be obtained (the mass of MMS was also used as the standard to be recorded as 2 mg of sample). If MMS/MSCE carries a fluorescent label, MMS/MSCE/CAT also carries the corresponding fluorescence, and is stored in a refrigerator at -20°C, protected from light.

**Characterization**

TEM images were obtained JEM-2100 (TEM, Hitachi, Japan). Zeta potential was recorded by a Zetasizer (Nano-Z, Malvern, UK). All the fluorescence images were taken by Confocal laser scanning microscopy (CLSM, HP Apo TIRF 100X N.A. 1.49, Nikon, Ti-E-A1R, Japan). Nanoparticle tracking analysis (NTA) of exosomes were detected by Particle Metrix’ ZetaView (Particle Metrix, Germany). Exosomal markers were detected by Western blot. Fourier transform infrared (FTIR) spectra were acquired on the Cary 5000 FTIR spectropho-tometer (Varian, USA). Catalase activity in different samples was determined by catalase activity assay kit (CheKine™ Micro Catalase Activity Assay Kit, Abbkine Scientific Co., Ltd., China). Absorbance data were obtained by microplate reader (Multiskan FC, Thermo Fisher Instruments, Inc., USA). The magnetic strength of different samples is measured by vibrating sample magnetometer (VSM-7404, Lake Shore Cryotronics, Inc., USA). The kinematic behavior of the nanomotor was captured by inverted fluorescence microscopy (MF53-N, Guangzhou Microshot Technology Co., Ltd., China). In vitro MRI characteristics of the nanomotor was determined by 9.4-T small animal magnetic resonance imaging machine (BioSpec 94/20 USR, Bruker Corporation, USA). The exact concentration of Fe in the samples was determined by ICP-MS (MC-ICP-MS, Thermo Fisher Instruments, Inc., USA).

**Isolation and Culture of SMCs from Elastase Injury-induced Rat AAAs (EaRASMCs)**

Primary EaRASMCs were isolated from aneurysmal aorta explants obtained from adult male Sprague-Dawley rats at 14 days post-AAA induction via elastase infusion ^[2]^. Similar to methods previously reported in the literature^[3]^, the rats were anesthetized by intraperitoneal injection of 2% sodium pentobarbital and immersed in 75% alcohol for 10 min. The animals were then fixed and sterilized, the abdominal skin was cut to fully expose the abdominal vessels, the abdominal aorta was separated from the inferior vena cava, the peripheral branches were cut, and the area of aneurysm formation was cut up and down in a sterile Petri dish and washed by soaking in sterile PBS buffer. Use ophthalmic forceps and scalpel to scrape the outer membrane, move gently to avoid mechanical strain to make cell damage, then cut the lumen and gently scrape the inner membrane. The mesentery was placed in DMEM high glucose medium containing 20% FBS, and the mesentery was cut with ophthalmic scissors so that the tissue was in 1 mm^2^ pieces. The tissue suspension was collected in a 15 mL centrifuge tube and centrifuged at 800 rpm for 5 min to remove the supernatant. Take 2 mL of whole serum to resuspend the tissue to prepare the suspension, transfer the suspension evenly to T25 cell culture flasks, shake gently to evenly distribute the tissue fragments, use a 1 mL lance tip to aspirate off most of the serum and turn the flasks over without dropping the tissues, place the flasks at 37°C in an incubator for 3 - 6 h, and observe that the tissues were drying out and sticking to the bottom of the flasks, then turn the flasks over and add 2 mL of DMEM high glucose containing 20% FBS gently, and leave it to stand for a few minutes. Static culture was maintained for 7 days until observation of cells reaching 90% confluency, indicating readiness for passaging. The primary cells were proved to be VSMCs by immunofluorescence staining with α-SMA. Primary arterial VSMCs from normal rats were extracted and verified using the same method. Cells used in experiments were of passages 2 - 6.

**Assessment of** **Hydrogen Peroxide Content in Cells after Inflammation Stimulation**

EaRASMCs (5 × 10^4^ cells) were inoculated in cell culture dishes and adhered to the wall overnight, and then stimulated using TNF-α (10 ng mL^-1^) and IL-1β (10 ng mL^-1^) for 0, 4, 8, 12, and 24 h, respectively. The hydrogen peroxide content of cells was determined according to the protocol of the Hydrogen Peroxide Content Assay Kit (CheKine™ Micro Hydrogen Peroxide Assay Kit, Abbkine Scientific Co., Ltd., China).

**Motion Capture and Analysis**

EaRASMCs (5 × 10^4^ cells) were inoculated in cell culture dishes and adhered to the wall overnight. Then TNF-α and IL-1β was added for 24 h to induce inflammatory stimulation. Cy5-labeled samples (20 μL, 1 mg mL^-1^) were then added to the pre-stimulated EaRASMCs and normal VSMCs environments, respectively. The motion was recorded by inverted fluorescence microscope. Then, the software of Image J was used to analyze the trajectories of 10 randomly selected particles and calculate the average velocity and velocity distribution histogram.

**Dynamic Chemotaxis Behavior of Nanomotors in a Micro-fluidic Channel**

The chemotaxis assay of nanomotors was performed using a Ψ-shaped glass substrate microchannel model, which is a microfluidic chip with a length of 22 mm, a width of 1.5 mm, a height of 300 μm, and a branching channel with a width of 500 μm (Figure S6). Specific experiments were performed as follows: EaRASMCs and VSMCs (5 × 10^4^ cells) were inoculated in cell culture dishes and adhered to the wall overnight. EaRASMCs were stimulated by TNF-α and IL-1β for 24 h. VSMCs were untreated. After discarding the upper layer of medium, the cells were washed three times with PBS buffer, and 1 mL of RIPA lysate was added to each, and the lysates were fully lysed and then diluted by adding 3 mL of PBS buffer. The VSMCs cell lysate dilution was buffer I; the sample solution labeled by Cy5 was buffer II; the VSMCs cell lysate dilution was buffer III-1, and the EaRASMCs cell lysate dilution was buffer III-2. The injection flow rate was controlled at 0.6 mL h^-1^. A continuous video was taken for 30 s (1 fps) using an inverted fluorescence microscope at a position near the outlet. Fluorescence intensity perpendicular to the flow direction was measured using ImageJ software to analyze the chemotactic displacement of the nanomotors.

**Cell Activity Test**

The microplate reader (Multiskan FC, Thermo Fisher instruments Co., Ltd., US) were used to analyze the cell viability. About 1 × 10^4^ cells well^-1^ of EaRASMCs were seeded in a 96-well plate and cultured for 12 h. The groups requiring stimulus treatment were added TNF-α and IL-1β and further incubated at 37°C for 24 h. Further, different concentration (0, 10, 20, 50, 100, 200 µg mL^-1^) of MMS/MSCE, MMS/CAT or MMS/MSCE/CAT was added into each well in triplicate. Then, the cell viability was quantified by using the CCK-8 assay.

**Cellular Uptake Analysis**

For qualitative analysis of cellular uptake, EaRASMCs (5 × 10^4^ cells) were seeded into the culture dishes to adhere to the wall. After then, the groups requiring stimulus treatment were added TNF-α and IL-1β and further incubated at 37°C for 24 h. Then, Cy5 labeled different nanoparticles (200 μg mL^-1^) or MSCE (1.04 μg mL^-1^) was added into the above culture dishes for 24 h in the dark. Subsequently, the treated cells were then slowly washed with PBS, and stained with DiO (10 μM, dispersed in DMSO) and DAPI (5 µg mL^-1^) for 15 min. After washing gently with PBS, the fluorescence images of EaRASMCs were collected with CLSM.

For quantitative analysis of cellular uptake, EaRASMCs (1 × 10^5^ cells) was incubated for overnight in a 6-well plate. After then, the groups requiring stimulus treatment were added TNF-α and IL-1β and further incubated at 37°C for 24 h. Then, Cy5 labeled different nanoparticles (200 μg mL^-1^) or MSCE (1.04 μg mL^-1^) were added into the above culture dishes for 24 h in the dark. Subsequently, the supernatant and the cells were collected, respectively, following by the treatment with washing and centrifugation (1500 rpm, 5 min). Then, 250 µL of cell lysate was added to completely lysate the treated cells. Finally, the content of Cy5 (representing captured samples) was detected at 649 nm by fluorescence spectrophotometer (F-4600, Hitachi, Japan).

$$\text{Cellular uptake efficiency }\left( \text{\%} \right)\text{=}\frac{\text{Fluorescence intensity of Cy5 from the lysed cell}\text{s}}{\text{Fluorescence intensity of Cy5 from supernatant and the lysed cells}}\text{×100}$$

**Immunofluorescence Detection of Elastic Matrix**

Elastic matrix protein expression in control and treated EaRASMC cultures after treatment was visualized using IF. EaRASMCs were cultured for a total of 21 days in glass bottom chamber slides (NuncTM Lab-TekTM II Chamber SlideTM System, Thermo Fisher Scientific) with cytokine injury and treatments performed at day 7 and 14 post-seeding. At the end point, the cell layers were washed with PBS and fixed in ice-cold methanol (20 min). Cells were blocked in PBS containing 5% v/v goat serum (Thermo Fisher Scientific; 20 min) and immunolabeled with primary antibodies (Elastin, sc-58756, Santa Curz, 1:100) overnight at 4°C. They were subsequently incubated with fluorescently conjugated secondary antibody for 1 h at room temperature. Nuclei were stained with a mounting medium containing DAPI (G1012, Solarbio Science & Technology Co., Ltd., China). The same concentration of rabbit isotype control was used as a negative control. Subsequently, the sections were scanned with CLSM. ImageJ software was used to analyzed the fluorescence intensity.

**ELISA Analysis of Desmosine in EaRASMCs**

EaRASMCs were cultured for a total of 21 days with cytokine injury and treatments performed at day 7 and 14 post-seeding. At the end point, the cell layers were washed with PBS. The concentration of desmosine in cell layers were quantified using ELISA following the manufacturer’s instructions (rat desmosine ELISA kit, Shanghai Enzyme Linked Biotechnology Co., Ltd, China).

**Quantification of the Intracellular ROS Generation in VSMCs**

EaRASMCs (5 × 10^4^ cells) were inoculated in cell culture dishes and adhered to the wall overnight. The cell culture medium was replaced with fresh medium containing different nanoparticles (200 μg mL^-1^) or MSCE (1.04 μg mL^-1^) and incubated for 24 h. After stimulation with TNF-α and IL-1β for 24 h, the medium was removed and 1 mL of fresh medium containing 10 μM DCFH-DA was added to each well, followed by 30 min incubation at 37°C. Subsequently, cells were washed 3 times with serum-free medium, and observed by fluorescence microscopy. Cellular fluorescence intensity was normalized using ImageJ software.

In addition, ROS levels in EaRASMCs were quantified by luciferase labeling. EaRASMCs (1 × 10^4^ cells) were inoculated in 96-well plates and cultured overnight. The cells were stimulated with TNF-α and IL-1β for 24 h. After switching to DMEM containing different nanoparticles (200 μg mL^-1^) or MSCE (1.04 μg mL^-1^) for 24 h, the cells were stained with the ROS probe DCFH-DA. Fluorescence intensity at Ex/Em=488/525 nm was detected using a fluorescence zymography.

**Western Blot Analysis**

Total proteins were extracted using RIPA lysates (Beyotime China, P0013B) containing 1% protease inhibitor cocktail (Beyotime China, P1005). The proteins were separated by an SDS-PAGE gel and then transferred to PVDF membranes. After incubation in 5% BSA blocking buffer for 60 min, the membranes were incubated overnight at 4°C with primary antibodies (TSG101 Rabbit mAb, Proteintech, 28283-1-AP; CD63 Rabbit mAb, Proteintech, 25682-1-AP; CD73 Rabbit mAb, Proteintech, 12231-1-AP; Vimentin Rabbit mAb, Proteintech, 10366-1-AP; MMP-2 Mouse mAb, Santa Cruz, sc-53630; LOX Mouse mAb, Santa Cruz, sc-21735; TIMP-2 Mouse mAb, Santa Cruz, sc-21735; PI3K Rabbit mAb, CST, #4992; p-PI3K Rabbit pAb, Proteintech, AP0427; Akt Mouse mAb, Santa Cruz, sc-81434; p-Akt Rabbit pAb, Proteintech, A18120; β-Actin Mouse mAb, CST, #3700). Thereafter, the membranes were incubated with corresponding HRP-conjugated IgG secondary antibodies (HRP-labeled Goat Anti-Rabbit IgG (H+L), WB2177, WELLBI Shanghai, 1:5000; HRP-labeled Goat Anti-Mouse IgG (H+L), WB3176, WELLBI Shanghai) for 1 h at room temperature. Protein bands were detected by a digital chemiluminescence system (Bio-Rad USA) and normalized using the ImageJ software.

**ELISA Analysis of Inflammatory Cytokines in RAW264.7 Macrophages**

The RAW264.7 cells (5 × 10^5^ cells per well) were seeded in 6-well plates and incubated overnight. Then, cells were stimulated by LPS (100 ng mL^-1^) for 24 h and then treated with different nanoparticles (200 μg mL^-1^) or MSCE (1.04 μg mL^-1^) for 24 h. Culture supernatants was collected and stored at −80°C until use. The concentration of TNF-α and IL-1β in culture supernatants were quantified using ELISA following the manufacturer’s instructions (mouse TNF-α ELISA kit, ExCell Biotech Co., Ltd, China; mouse IL-1β ELISA kit, ExCell Biotech Co., Ltd, China).

**Cellular Transcriptome Sequencing** **Analysis**

To investigate the differences in gene expression of EaRASMCs after MMS/MSCE/CAT treatment, transcriptome sequencing was utilized. EaRASMCs (5 × 10^4^ cells) were inoculated into culture dishes overnight, and then cells were stimulated with TNF-α and IL-1β for 24 h. Cell culture medium was replaced with fresh medium containing MMS/MSCE/CAT (200 μg mL^-1^) and incubated for 24 hours. The control group was not treated. Total RNA was extracted and examined by adding Trizol reagent to the cell samples from the nanomotor treatment and control groups. Then sequencing libraries were constructed and quality control was performed on the constructed libraries. Sequencing was then performed using the Illumina NovaSeq 6000 platform. After sequencing, we obtained the raw data and entered the bioinformatic analysis process, which included data quality control, comparative analysis, expression analysis, differential expression analysis and enrichment analysis.

**Animals**

Male Sprague-Dawley rats (150 - 160 g) were purchased from Shanghai SLAC Laboratory Animal Co. Ltd. (Shanghai, China). All animal experiments were performed under the guidelines of the Animal Care Committee of Nanjing Normal University (IACUC-20201101−1, Nanjing, China). All animals were housed in standard cages under suitable light, temperature, and humidity environment, with ad libitum access to food and water. Animals were acclimatized to the laboratory for at least 5 days before further experiments.

**Establishment of the AAA Model in Rats**

AAA in rats was established according to the previously reported methods^[4]^. In brief, rats were anesthetized by inhalation of 1-2% isoflurane. The infrarenal abdominal aorta was isolated from the left renal vein to the aortic bifurcation in a midline open laparotomy, and the beginning and distal portions of the isolated aorta were temporarily ligated with 4-0 sutures. A PE-10 tube was then introduced into the infrarenal aorta, and 0.27 mL of porcine pancreatic elastase (0.27 units, type I; Sigma-Aldrich, USA) in PBS was continuously infused for 0.9 hours with a syringe pump. At the end of the infusion, the aorta was closed and the PE-10 tube was removed.

**In Vivo Imaging**

To assess the in vivo targeting ability, different samples labeled with Cy5 were injected i.v. separately into AAA rats. AAA rats were randomly divided into 4 groups (n = 3) and subjected to the following treatments: tail vein injections of MSCE (13 μg kg^-1^, 200 μL), MMS/MSCE/CAT (2.5 mg kg^-1^, 200 μL), and tail vein injections of MMS/MSCE (2.5 mg kg^-1^, 200 μL) or MMS/MSCE/CAT (2.5 mg kg^-1^, 200 μL) and then a magnetic field was applied to the aneurysm area for 1 h. Rats in the control group were subjected to i.v. injection of PBS. 8 h later, the thoracic aorta, abdominal aorta, and bilateral iliac arteries were harvested together for in vivo imaging analyses via an in vivo imaging system (IVIS Spectrum, PerkinElmer).

Cy5-labeled MMS/MSCE/CAT (2.5 mg kg^-1^, 200 μL) were injected into AAA rats through the tail vein and then a magnetic field was applied to the aneurysm area for 1 h. 6 h, 12 h and 24 h later, the thoracic aorta, abdominal aorta, and bilateral iliac arteries were harvested together for in vivo imaging analyses via an in vivo imaging system (IVIS Spectrum, PerkinElmer).

**Biodistribution**

As described above, Cy5-labeled different samples were injected into AAA rats through the tail vein and a magnetic field was applied to the aneurysm region. After a certain period of time, the mice were executed to remove the major organs and the major organs (heart, liver, spleen, lung, and kidney) were lysed and ground for homogenization. The fluorescence intensity in the supernatant was measured by centrifugal separation. The percentage of the injected dose per gram of tissue (% ID/g) was calculated.

**In Vivo Therapeutic Effect Study**

To study in vivo efficacies of different samples in rats with AAA induced by elastase, male Sprague-Dawley rats (150 - 160 g) were randomly assigned into five groups. The abdominal aorta's maximum diameter was measured using digital color diagnostic ultrasound before and 14 days after elastase infusion to confirm successful modeling. Post-modeling, treatments were administered on days 0, 3, 6, 9, and 12: tail vein injections of PBS buffer (200 μL), MSCE (13 μg kg^-1^, 200 μL), MMS/MSCE/CAT (2.5 mg kg^-1^, 200 μL), and tail vein injections of MMS/MSCE (2.5 mg kg^-1^, 200 μL) or MMS/MSCE/CAT (2.5 mg kg^-1^, 200 μL) and then a magnetic field was applied to the aneurysm area for 1 h. Rats in the normal control group were subjected to i.v. injection of PBS (200 μL). On day 15 after treatments, the maximum diameter of abdominal aorta was again measured using digital color ultrasonography before euthanasia. Whole blood was collected via cardiac puncture at time of sacrifice. The abdominal aorta was dissected and frozen directly in OCT. Compound and sectioned at a thickness of 5 μm. Sections were stained with hematoxylin and eosin (H&E) and Verhoeff's-van Gieson (EVG) stain. Immunofluorescent staining with anti-elastin, anti-LOX, and anti- Fibrillin-1 antibodies was performed, along with in situ terminal transferase labeling technique (TUNEL staining) on the sections.

**Ultrasound Imaging**

The maximum diameter of the abdominal aorta was detected using small-animal color Doppler ultrasound (VINN06 LAB, Vino Technology Co., Ltd., China) in each group of Sprague-Dawley rats before modeling, 14 days after modeling, and 15 days of treatment. The rats were anesthetized with isoflurane gas, the limbs were immobilized, the abdomen was exposed, and the abdomen was carefully shaved with a razor. The parameters of the ultrasound machine were adjusted and preset to the color doppler flow imaging (CDFI) mode, and the intravascular diameters of the abdominal aorta were measured in the transverse and longitudinal sections, respectively.

**Hematoxylin and Eosin (H&E) Staining**

After euthanasia by overdose anesthesia, the abdominal aorta of rats was dissected, fixed in 4% paraformaldehyde solution and paraffin-embedded, and then sectioned at a thickness of 5 μm. Tissue sections were first baked overnight in a 37°C thermostat, followed by deparaffinization with xylene and hydration of the tissue with gradient alcohol; the sections were immersed in hematoxylin solution for 5 min, and then washed with distilled water for 5 min; the sections were lifted up and down in hydrochloric acid ethanol for about 3 s to be acidified, and then immersed in distilled water for 10 min; the sections were immersed in eosin solution for about 2 min; the sections were dehydrated by using gradient alcohol and clarified with xylene, and a drop of neutral resin was added to the sections, and the sections were covered with a coverglass to seal the slices and fully air-dried at room temperature; the changes in the structure of aneurysm tissues and the morphology of the cells were observed through a light microscope and photographed for recordings.

**Verhoeff's-van Gieson** **(EVG) Staining**

EVG staining was performed according to the protocol of the Collagen Fiber and Elastic Fiber Staining Kit (G1597, Solarbio Science & Technology Co., Ltd., China). First, the sections were heat-treated for 50 min and then dewaxed through xylene for 20 min and hydrated with gradient alcohol, the modified VG staining solution was added dropwise to the sections, and the sections were stained for 10 min and then washed in distilled water for 10 s. The Verhöeff staining work-up solution was added dropwise to the sections, and the sections were stained for 5 min and then washed in distilled water for 10 s. The Verhöeff differentiation solution was added dropwise to the sections to differentiate them for 10 s until the elastin fibers were clear Verhöeff differentiation solution was added to the sections for 10 s, until the elastic fibers were clear, and then washed with distilled water for 10 s. The sections were dehydrated with anhydrous ethanol and clarified with xylene, sealed with neutral gum, and then read and photographed under a microscope.

**Immunofluorescence Staining**

Tissues were frozen in OCT compound, and sectioned at 5 μm thickness. After being dried at room temperature for 15 min, sections were fixed in 4% paraformaldehyde for 10 min, permeabilized with 0.2% Triton X-100 for 10 min, blocked with 3% BSA for 30 min, and incubated with specific primary antibodies overnight at 4°C. They were subsequently incubated with fluorescently conjugated secondary antibody for 1 h at room temperature. Nuclei were stained with a mounting medium containing DAPI (G1012, Solarbio Science & Technology Co., Ltd., China). The same concentration of rabbit isotype control was used as a negative control. Subsequently, the sections were scanned with CLSM. ImageJ software was used to analyzed the fluorescence intensity. The following primary antibodies were used: Elastin (sc-58756, Santa Curz), LOX (sc-373995, Santa Curz), and Fibrillin-1 (ab231094, abcam).

**Terminal Deoxynucleotidyl Transferase dUTP Nick End Labeling (TUNEL) Assay**

The experiment was carried out on frozen sections according to the assay kit (C1090, Beyotime Biotech Inc, China) instruction. And the nucleus was labeled with DAPI. Fluorescence images were obtained by CLSM. TUNEL-positive cells are considered to be apoptotic cells.

**ELISA Analysis of Serum Desmosine in AAA Rats**

Whole blood was collected via cardiac puncture at time of sacrifice. The blood was allowed to coagulate at room temperature for 30 min and then centrifuged. Serum was collected and stored at −80°C until use. The desmosine concentration of serum samples were quantified using ELISA following the manufacturer’s instructions (rat desmosine ELISA kit, Shanghai Enzyme Linked Biotechnology Co., Ltd, China).

**In Vivo Toxicity Tests in Rats**

Blood samples from rats 24 hours after injection with different samples were collected for hematological analysis. At the end of 15 days of treatment, the rats were euthanized and the major organs such as heart, liver, spleen, lungs and kidneys were collected, fixed in 4% paraformaldehyde and stained for H&E after making tissue sections by paraffin embedding.

**In Vivo MRI**

The AAA rats were injected with MMS/MSCE (2.5 mg kg^-1^, 200 μL) or MMS/MSCE/CAT (2.5 mg kg^-1^, 200 μL) in the tail vein and then a magnetic field was applied to the aneurysm area. MRI scans of rats in each group were performed after 8 h of treatment using a 9.4-T small animal magnetic resonance imaging machine. Each animal was placed under continuous isoflurane anesthesia in the center of a whole-body coil (35 mm internal diameter), which was located in the scanner. The animals were connected to a respiratory rate monitor and the flow of anesthetic gas was continuously adjusted to maintain a respiratory rate of 60 breaths/min. The infrarenal region of the aorta 22 mm directly below the right renal artery was imaged. All magnetic resonance imaging experiments were performed on a 9.4 T small animal magnetic resonance imager. Images were acquired using the T_2_-RARE imaging sequence. Detailed magnetic resonance imaging parameters were as follows: repetition time (milliseconds)/echo time (milliseconds), 3700/30.0; image size, 256 × 256; slice thickness, 1.0 mm; slices, 38; and field of view, 25.6 mm × 19.2 mm. The acquisition time for acquiring each magnetic resonance imaging session was 8 minutes and 48 seconds. The acquired MRI data were transferred as DICOM images to RadiAnt DICOM Viewer for quantitative image analysis. Signal intensity (SI) loss on the lumen image of the abdominal aorta in T_2_ imaging was measured by manual tracing as described in the literature+. The aneurysmal region with the largest lumen area and around the unaffected portion of the normal aorta were manually traced as the region of interest (ROI). The formula for calculating the percentage reduction in standardized AAA SI was measured as follows:

$$\text{\%SI loss=100×}\left( \text{1-}\frac{\text{postcontrast normalized SI of AAA}}{\text{precontrast normalize}\text{d SI of AAA}} \right)$$

**Statistical Analysis**

All data are presented as means with standard errors. Statistical difference was evaluated using Student’s t test and the statistical difference in different groups via one-way ANOVA.


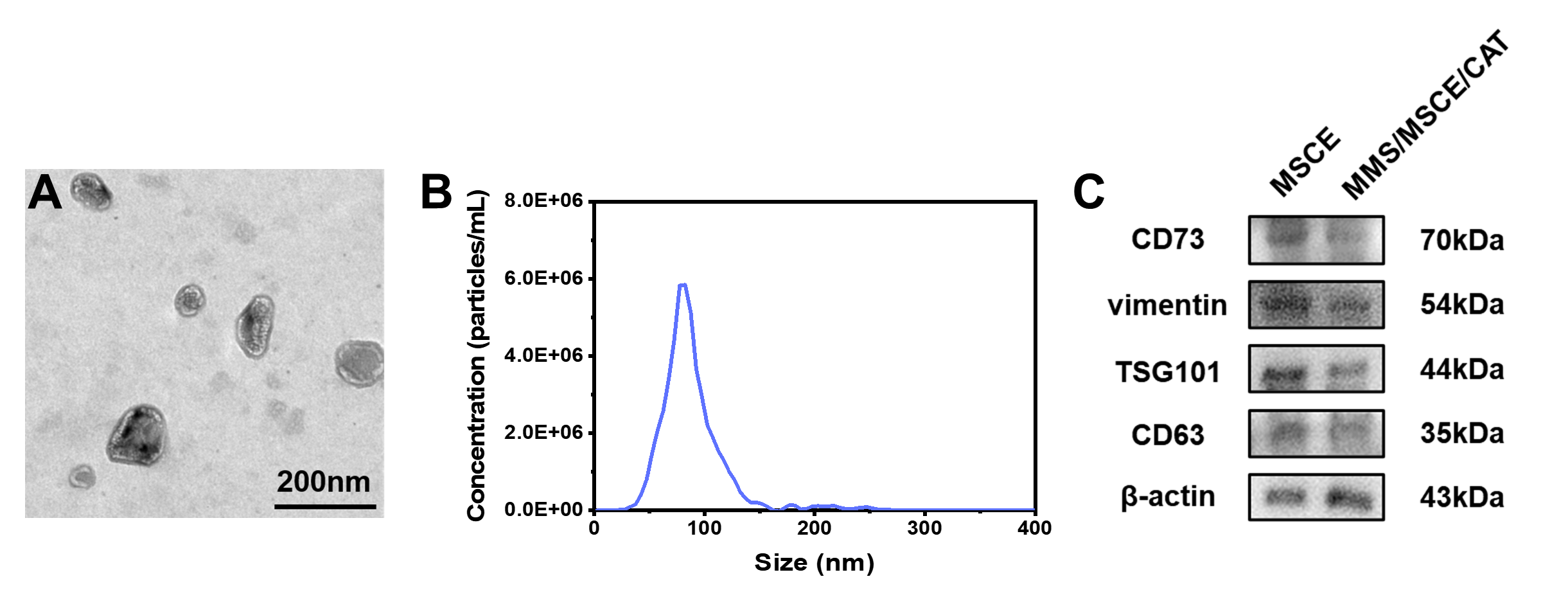


**Figure S1** (A) TEM image of MSCE, (B) NTA of MSCE, (C) WB analysis of exosome marker proteins in MSCE and MMS/MSCE/CAT.


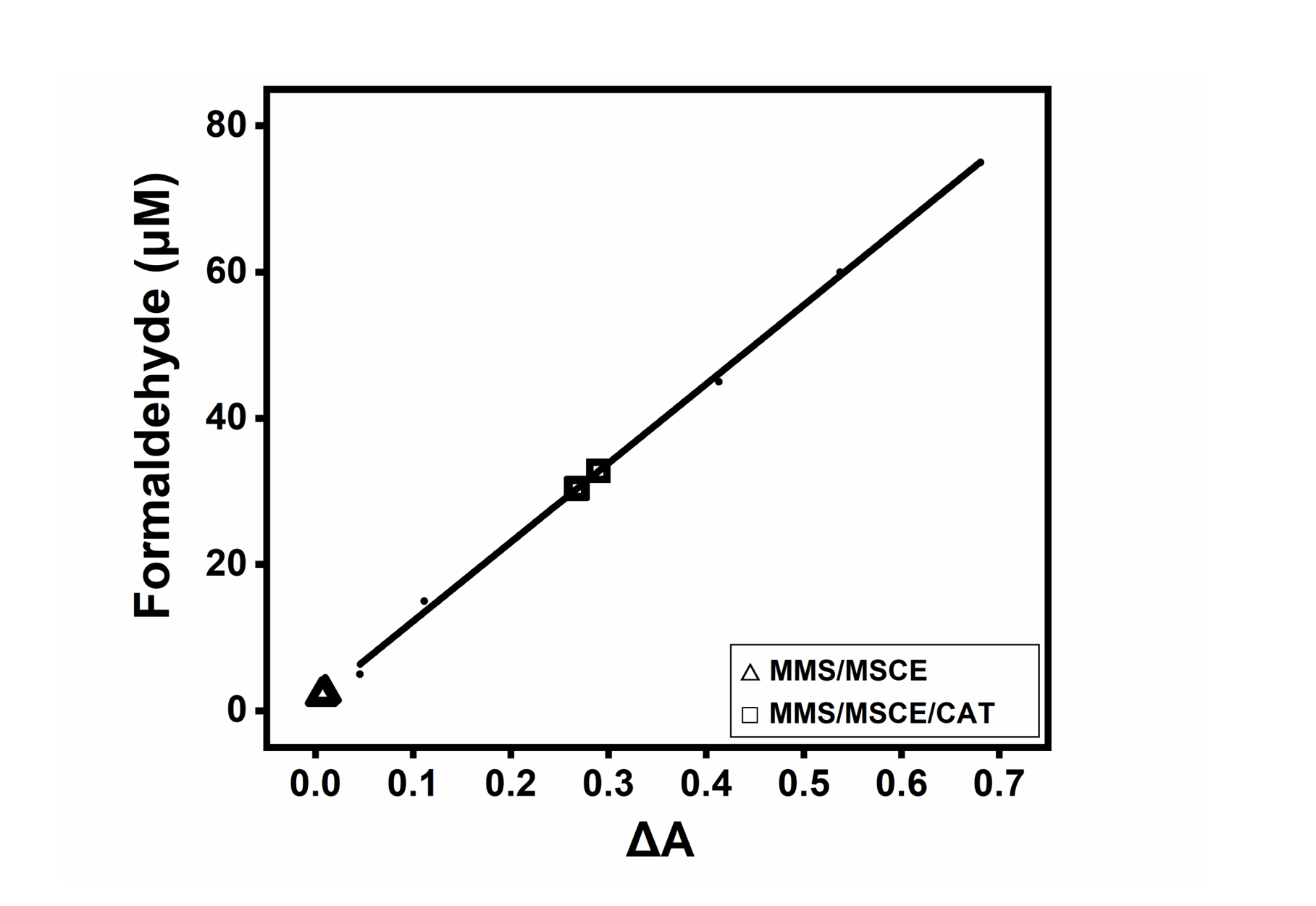


**Figure S2** Standard curve of catalase activity at room temperature and catalase activity of different nanoparticles (*n* = 3). Triangles indicate MMS/MSCE. Squares indicate MMS/MSCE/CAT.


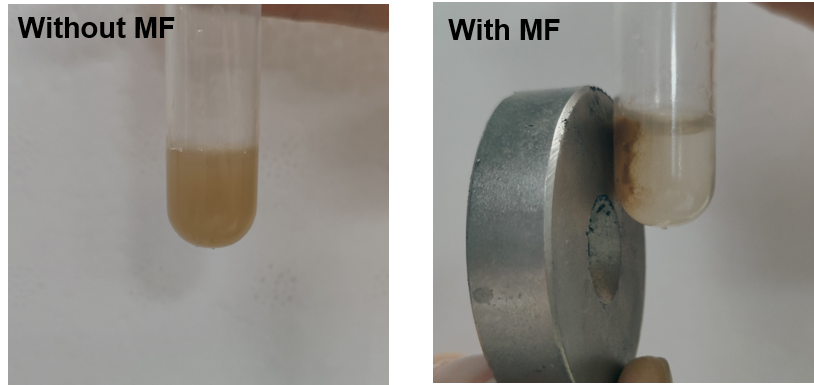


**Figure S3** Magnetic separation experiment.


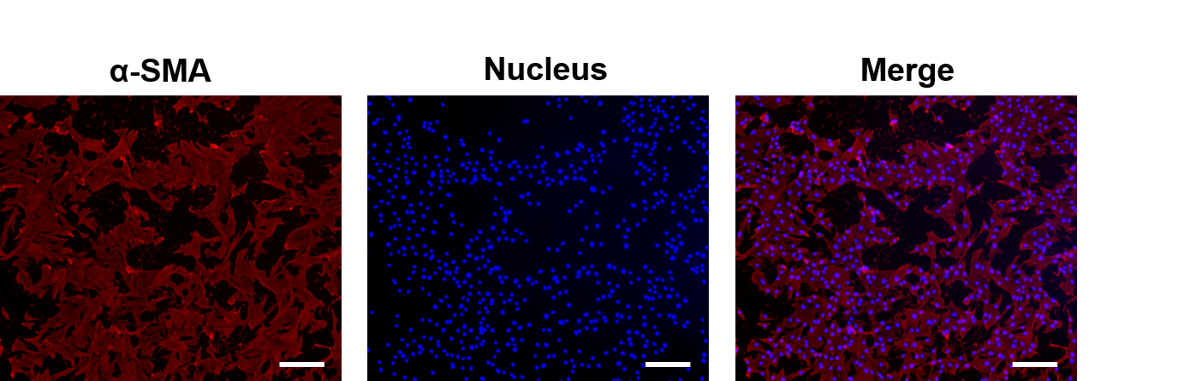


**Figure S4** Validation of primary arterial smooth muscle cells (Red fluorescence: α-SMA; Blue fluorescence: nucleus; Scale bar = 100 μm).


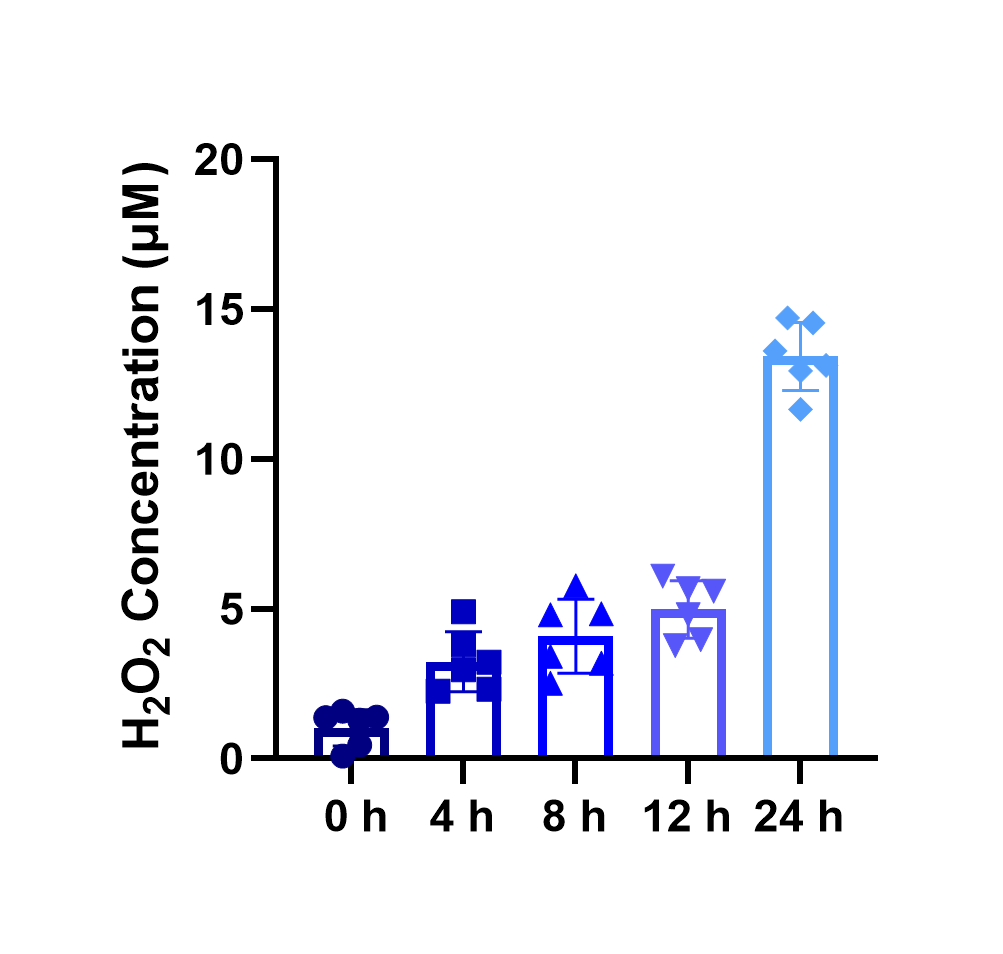


**Figure S5** Cellular H_2_O_2_ levels after 10 ng mL^-1^ TNF-α and 10 ng mL^-1^ IL-1β stimulation of EaRASMCs for 0, 4, 8, 12, and 24 h. Data are mean ± SD (*n* = 6).


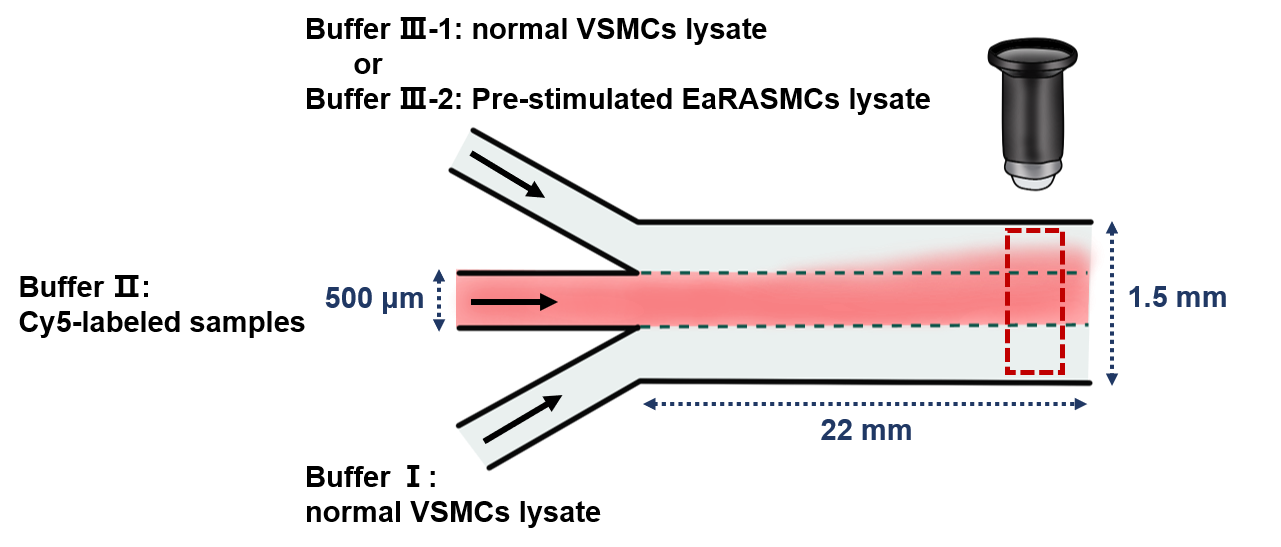


**Figure S6** Schematic illustration of dynamic microfluidic model with Cy5-labeled samples in buffer (II), prestimulated EaRASMCs lysate in buffer (III -2) and normal VSMCs lysate in buffer (I or III -1).


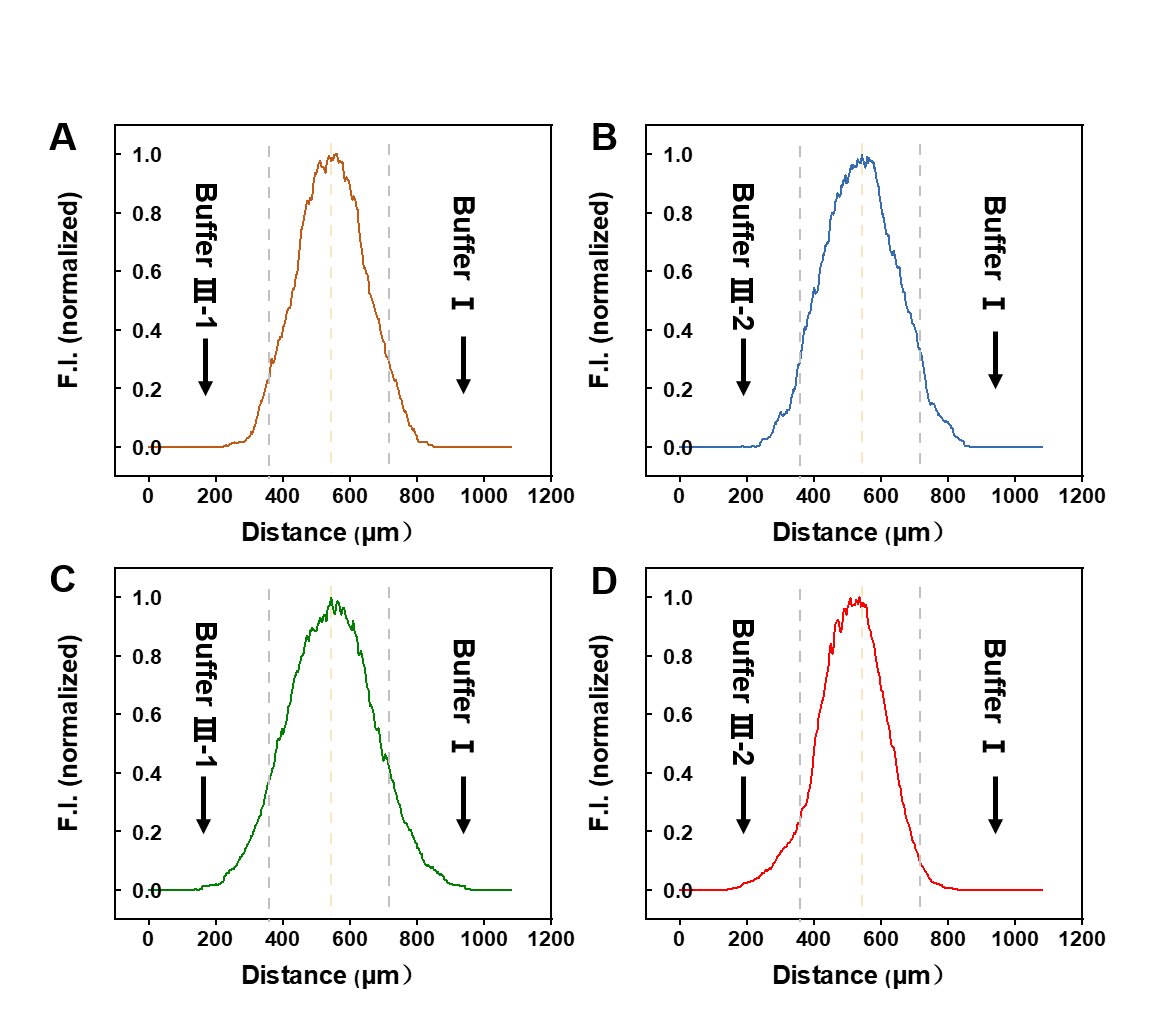


**Figure S7** Corresponding normalized fluorescence intensities of MMS/MSCE flow in the presence of (A) buffer I and buffer III-1, and (B) buffer I and buffer III-2 on two sides; Corresponding normalized fluorescence intensities of MMS/MSCE/CAT flow in the presence of (C) buffer I and buffer III-1, and (D) buffer I and buffer III-2 on two sides.


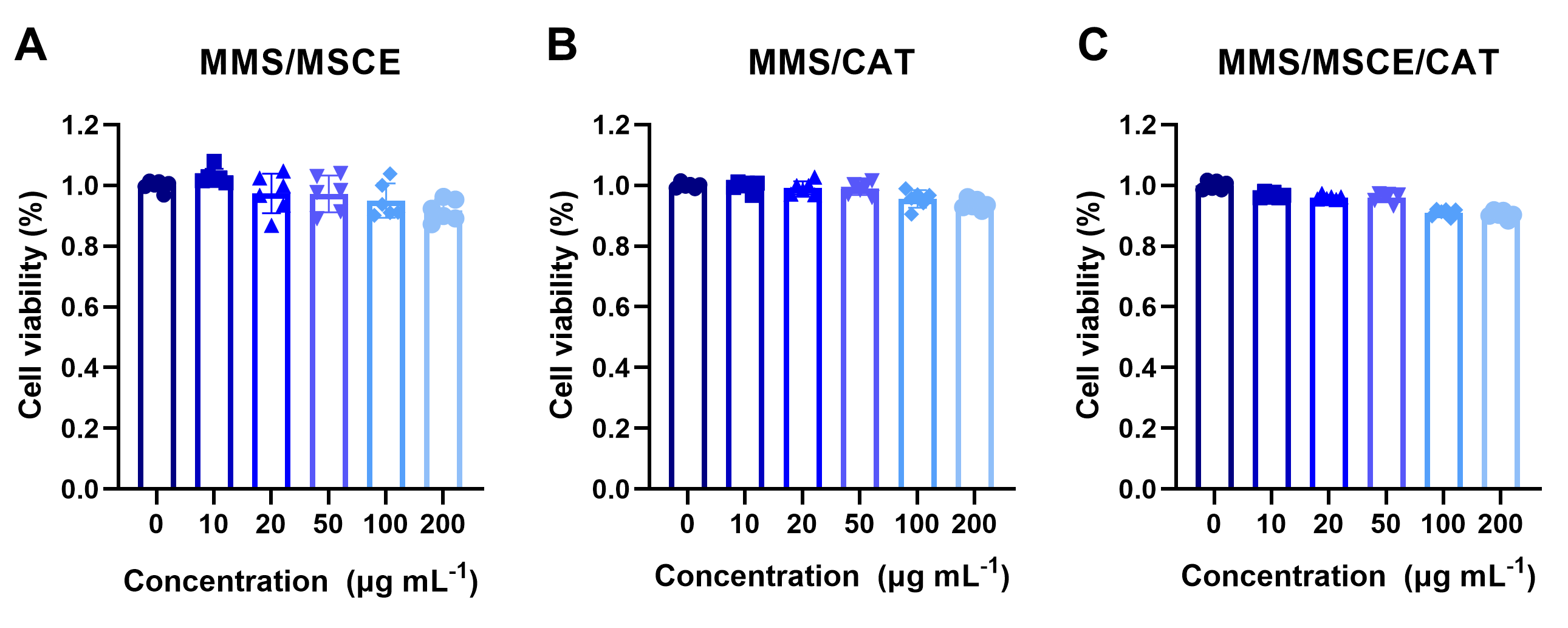


**Figure S8** Cell viability of EaRASMCs after incubation with various doses of different samples for 24 h. (A) MMS/MSCE; (B) MMS/CAT; (C) MMS/MSCE/CAT. Data are mean ± SD (*n* = 6).


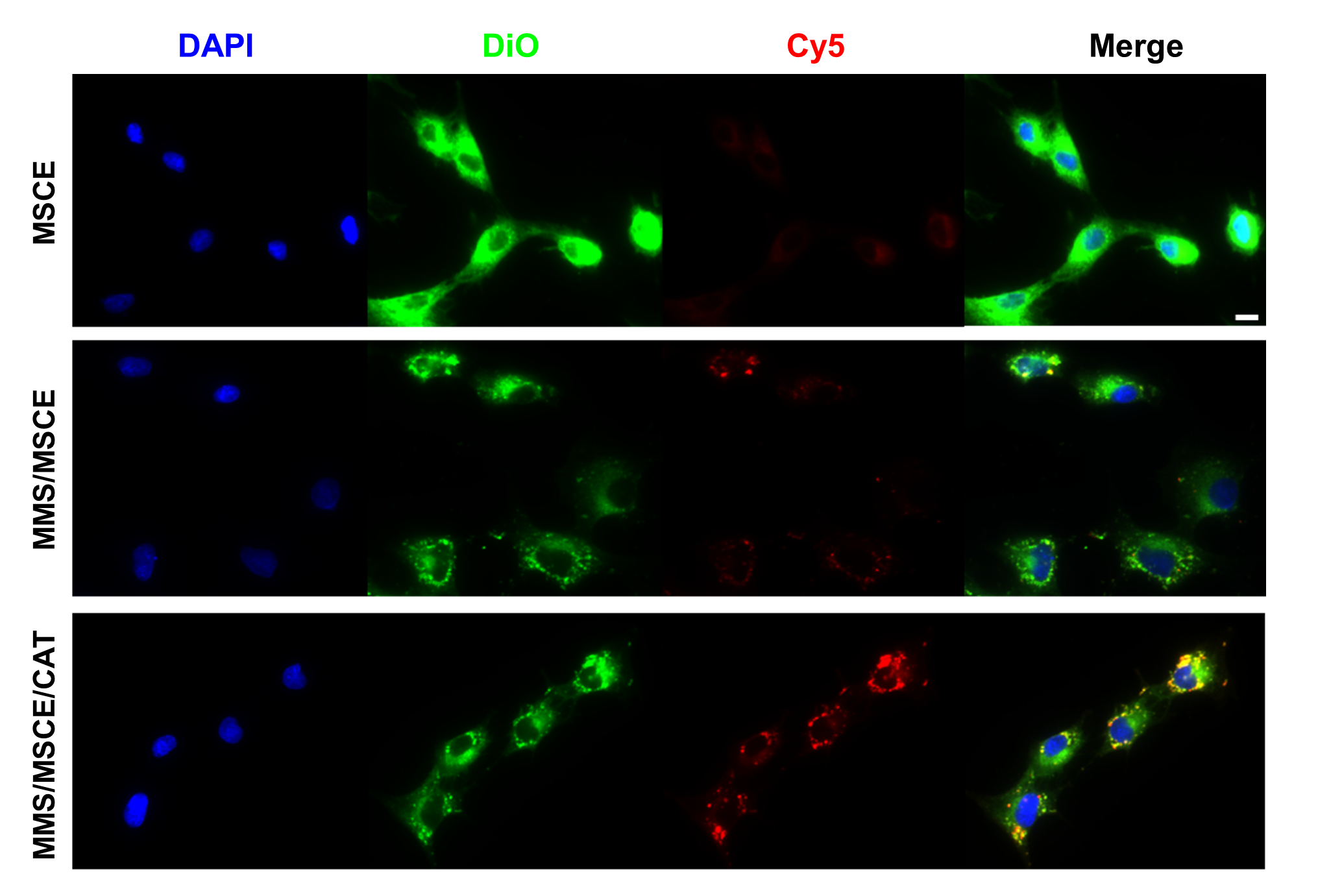


**Figure S9** Fluorescence images showing cellular uptake of MSCE, MMS/MSCE and MMS/MSCE/CAT in EaRASMCs. MSCE was stained with Cy5 (red), nuclei were stained with DAPI (blue) and cell membranes were stained with DiO (green). Scale bar = 10 μm.


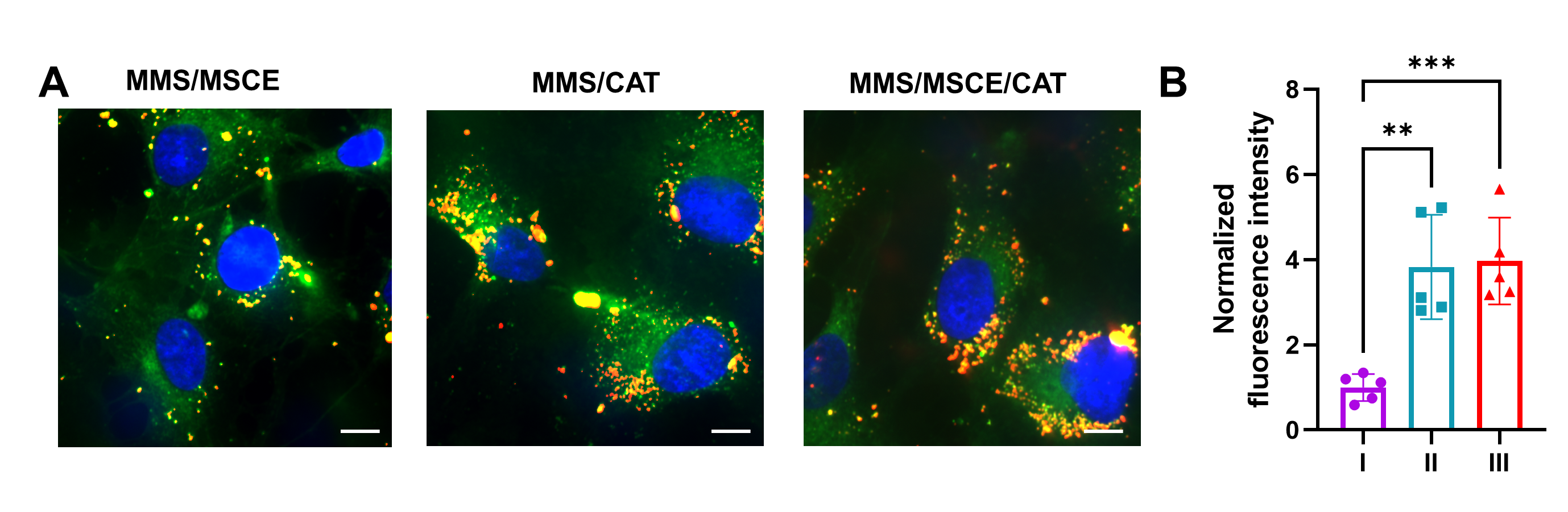


**Figure S10** (A) Representative fluorescence images of cellular uptake of MMS/MSCE, MMS/CAT and MMS/MSCE/CAT in pre-stimulated EaRASMCs (Scale bars = 10 μm) and (B) the corresponding normalized fluorescence quantification. Data are mean ± SD (*n* = 5). *P < 0.05, **P < 0.01, and ***P < 0.001, determined by one-way ANOVA.

**Figure S11** ELISA analysis of desmosine levels in EaRASMCs. Data are mean ± SD (*n* = 6). *P < 0.05, **P < 0.01, and ***P < 0.001, determined by one-way ANOVA.


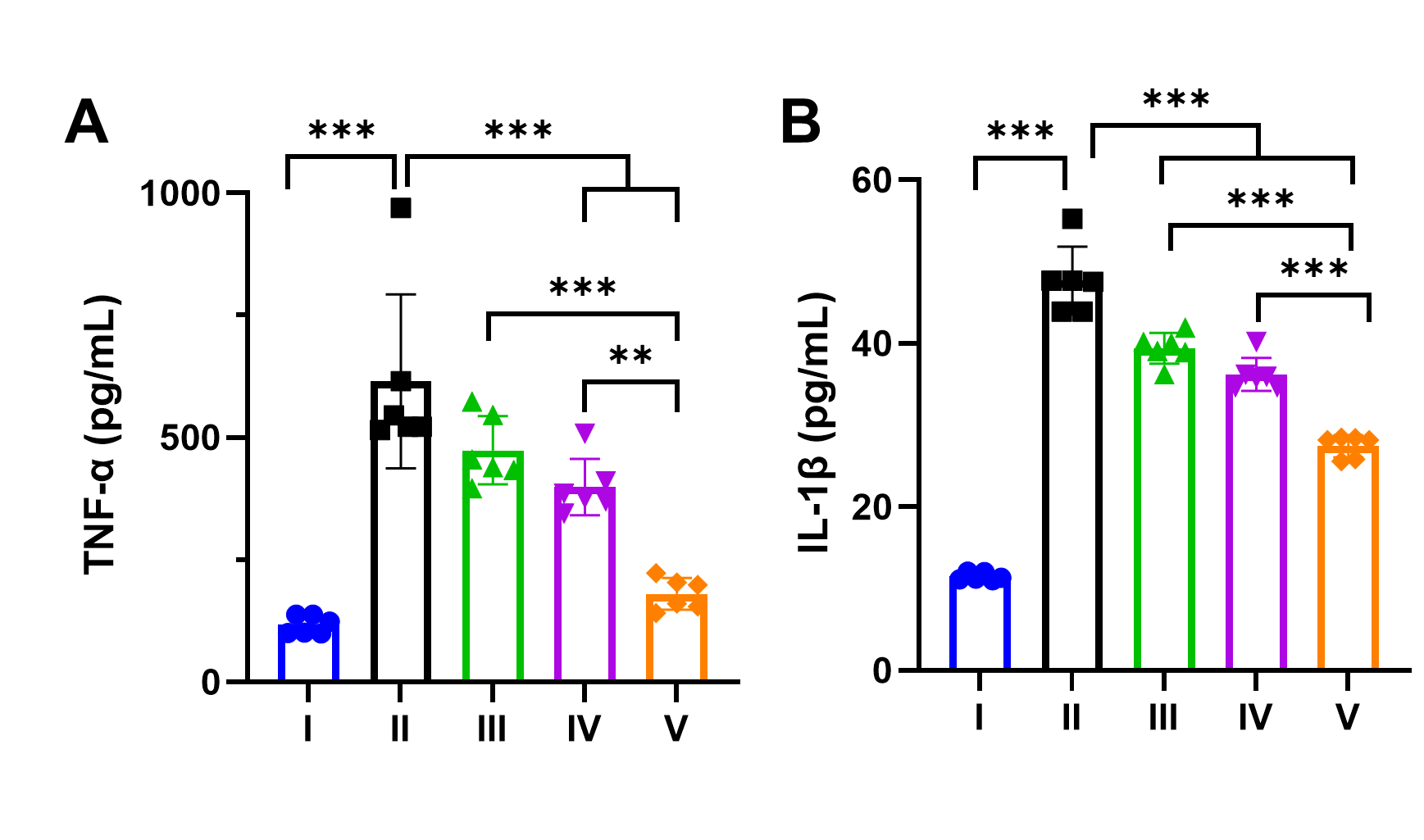


**Figure S12** TNF-α and IL-1β levels in culture supernatants collected from RAW264.7 cells cultures. Cells in the normal groups were treated with medium alone, while the model groups were stimulated with 100 ng mL^-1^ LPS. For three treatment groups, cells were pre-stimulation with LPS, followed by treatment with different samples (I: Normal, II: Model, III: MSCE, IV: MMS/MSCE, V: MMS/MSCE/CAT). Data are mean ± SD (*n* = 6). *P < 0.05, **P < 0.01, and ***P < 0.001, determined by one-way ANOVA.


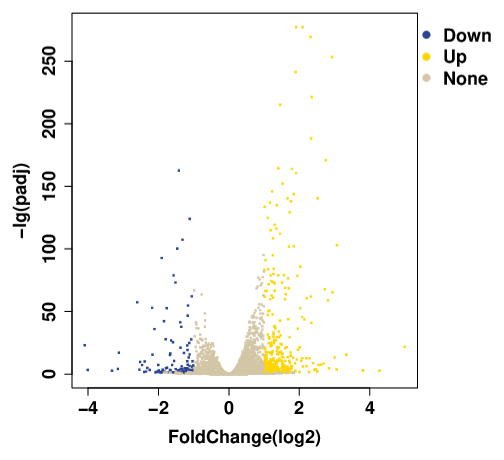


**Figure S13** Volcano plot of differentially expressed genes (Fold change ≥2.0 or ≤0.5, padj <0.05; Blue represents significantly down-regulated genes, yellow represents significantly up-regulated genes, and gray represents non-significantly different genes).

**
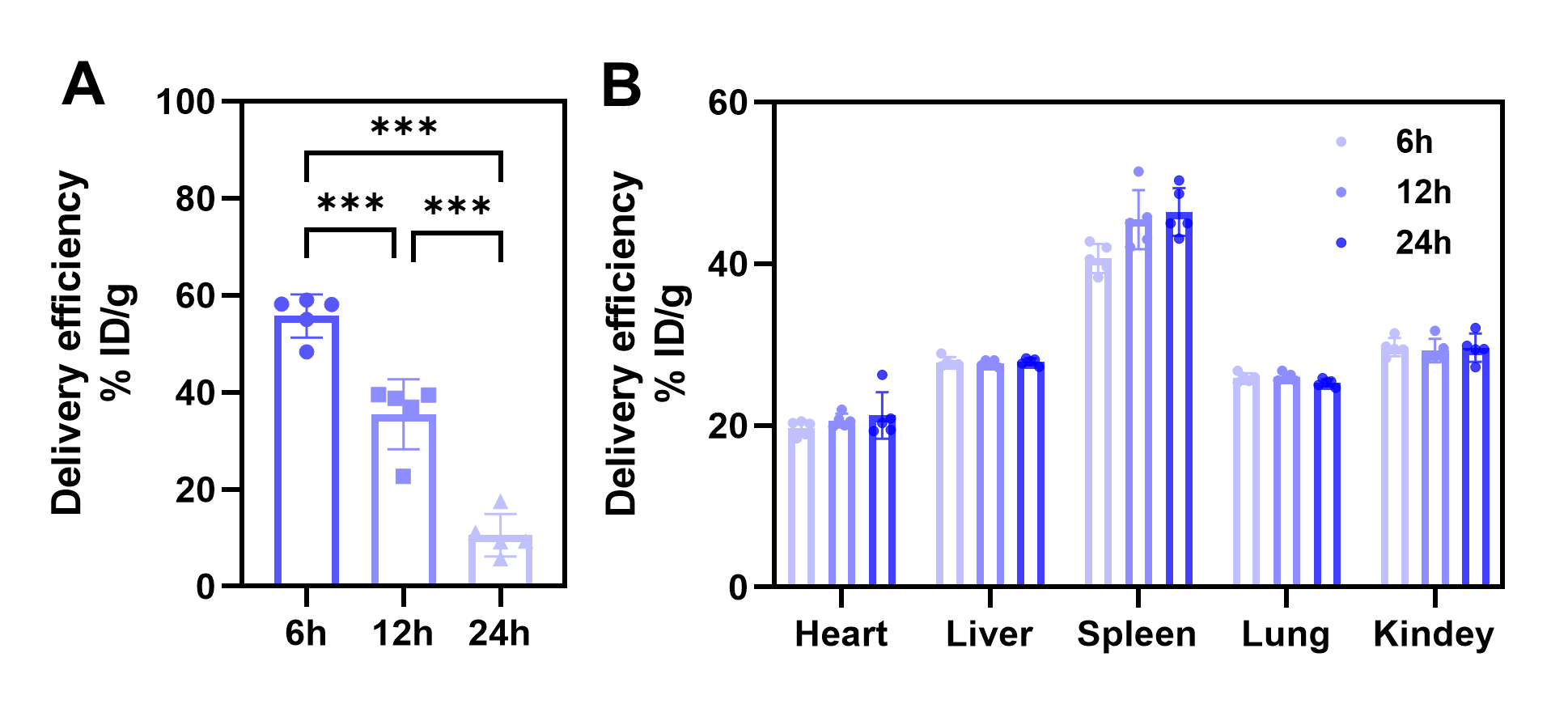
**

**Figure S14** Biodistribution of MMS/MSCE/CAT in (A) aneurysmal aortas and (B) other organs after intravenous injection for different times. MMS was labeled with Cy5 dye. Data are mean ± SD (*n* = 5). *P < 0.05, **P < 0.01, and ***P < 0.001, determined by one-way ANOVA.


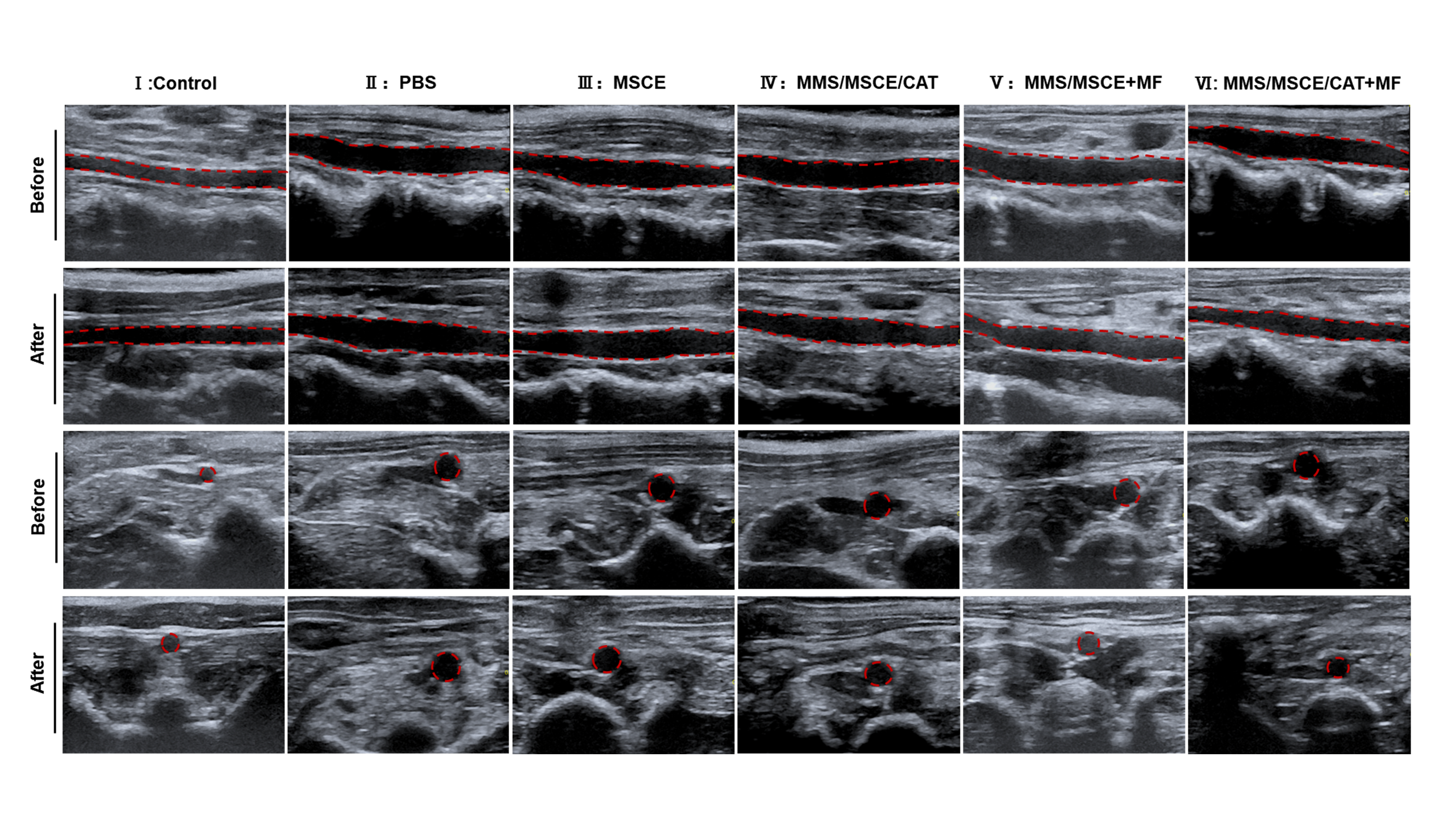


**Figure S15** Representative ultrasound images of the longitudinal and transverse sections of abdominal aortas for different groups.


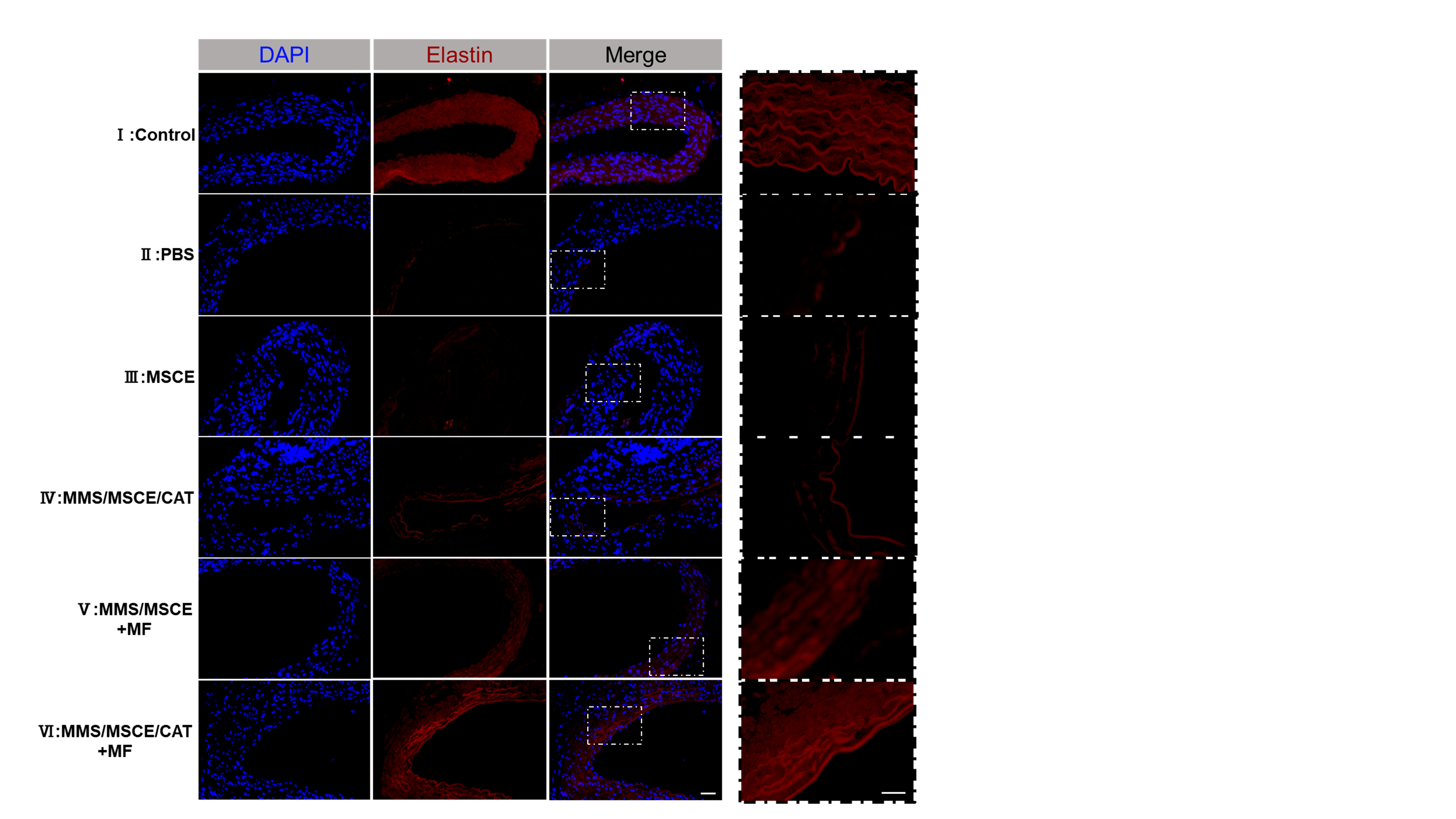


**Figure S16** Immunofluorescence images showing the expression of elastin (red) in the abdominal aorta. Scale bar = 50 μm; the corresponding enlarged views with a scale bar of 25 μm.


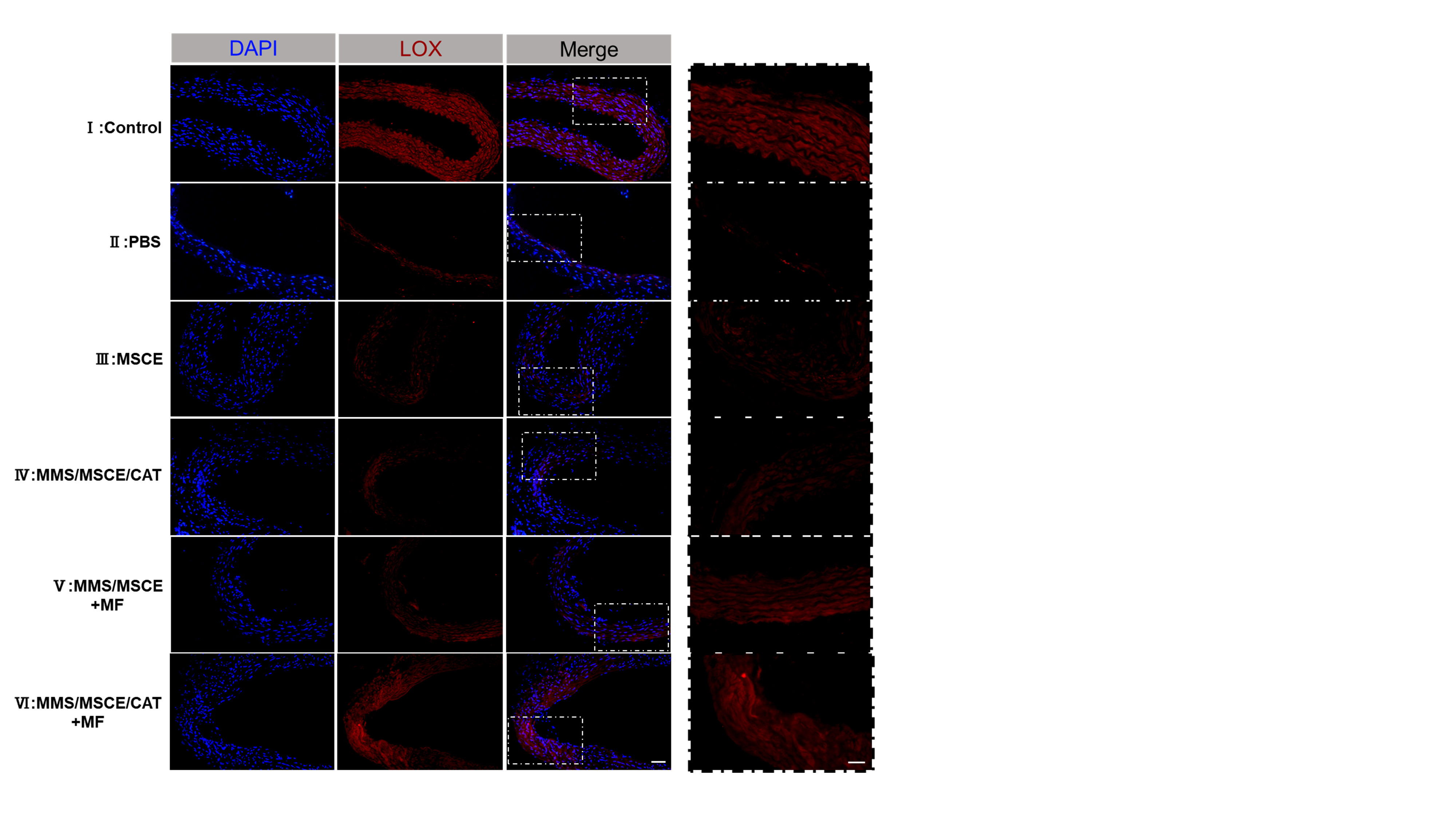


**Figure S17** Immunofluorescence images showing the expression of LOX (red) in the abdominal aorta. Scale bar = 50 μm; the corresponding enlarged views with a scale bar of 25 μm.


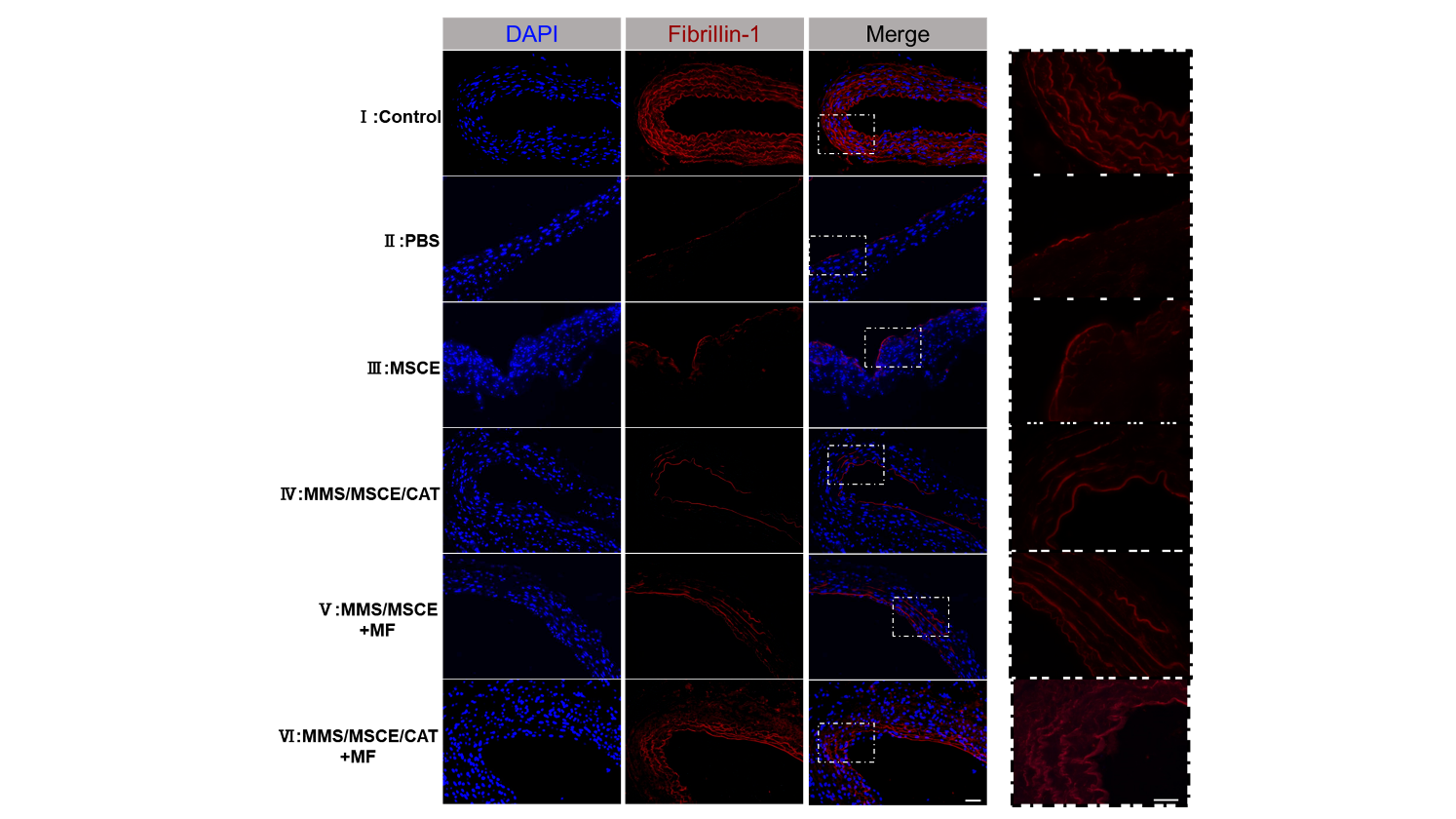


**Figure S18** Immunofluorescence images showing the expression of Fibrillin-1 (red) in the abdominal aorta. Scale bar = 50 μm; the corresponding enlarged views with a scale bar of 25 μm.


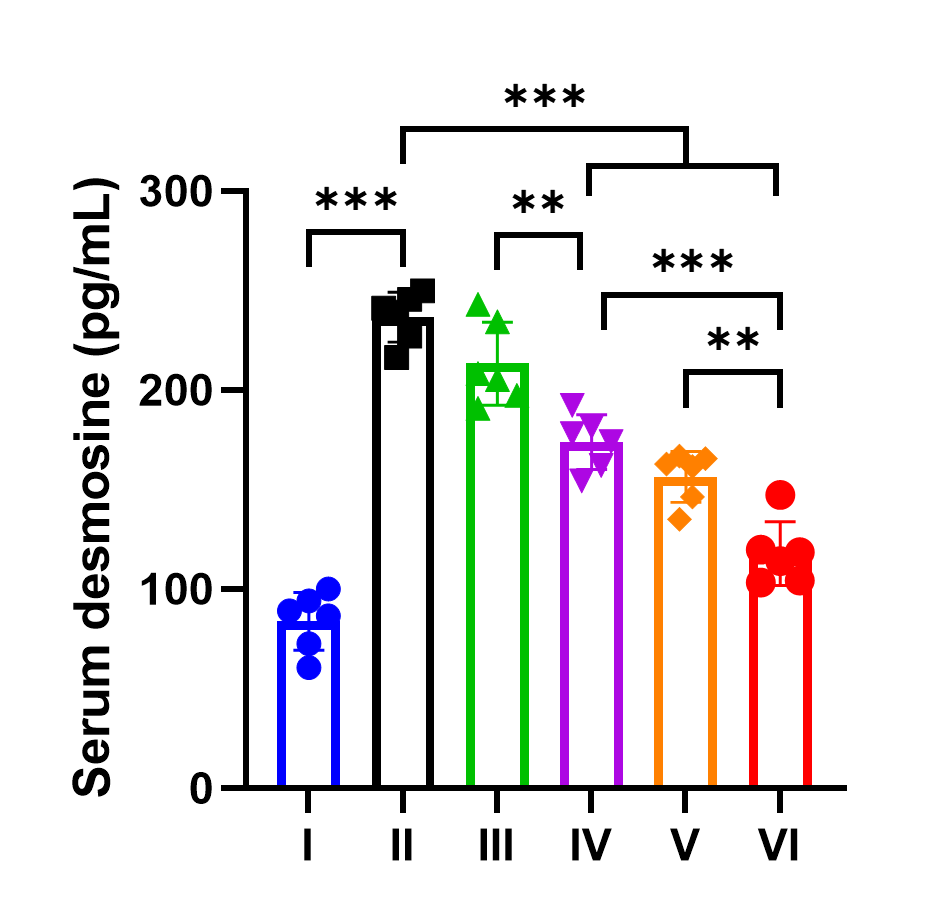


**Figure S19** ELISA analysis of serum desmosine levels in AAA rats subjected to different treatments. Data are mean ± SD (*n* = 6). *P < 0.05, **P < 0.01, and ***P < 0.001, determined by one-way ANOVA.


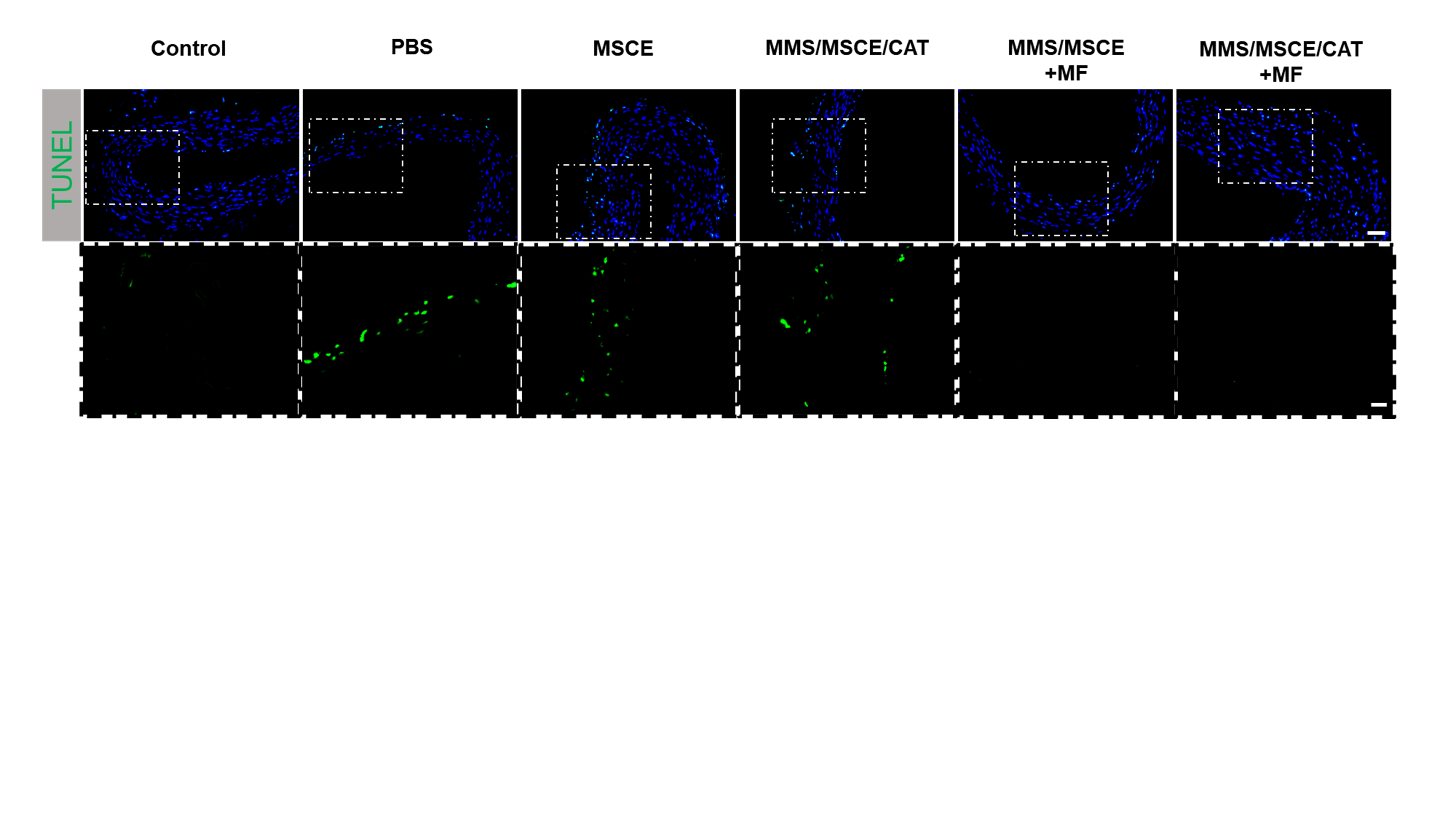


**Figure S20** Microscopy images of TUNEL-stained sections of aortic tissues from AAA rats subjected to different treatments. Scale bar = 50 μm; the corresponding enlarged views with a scale bar of 25 μm.


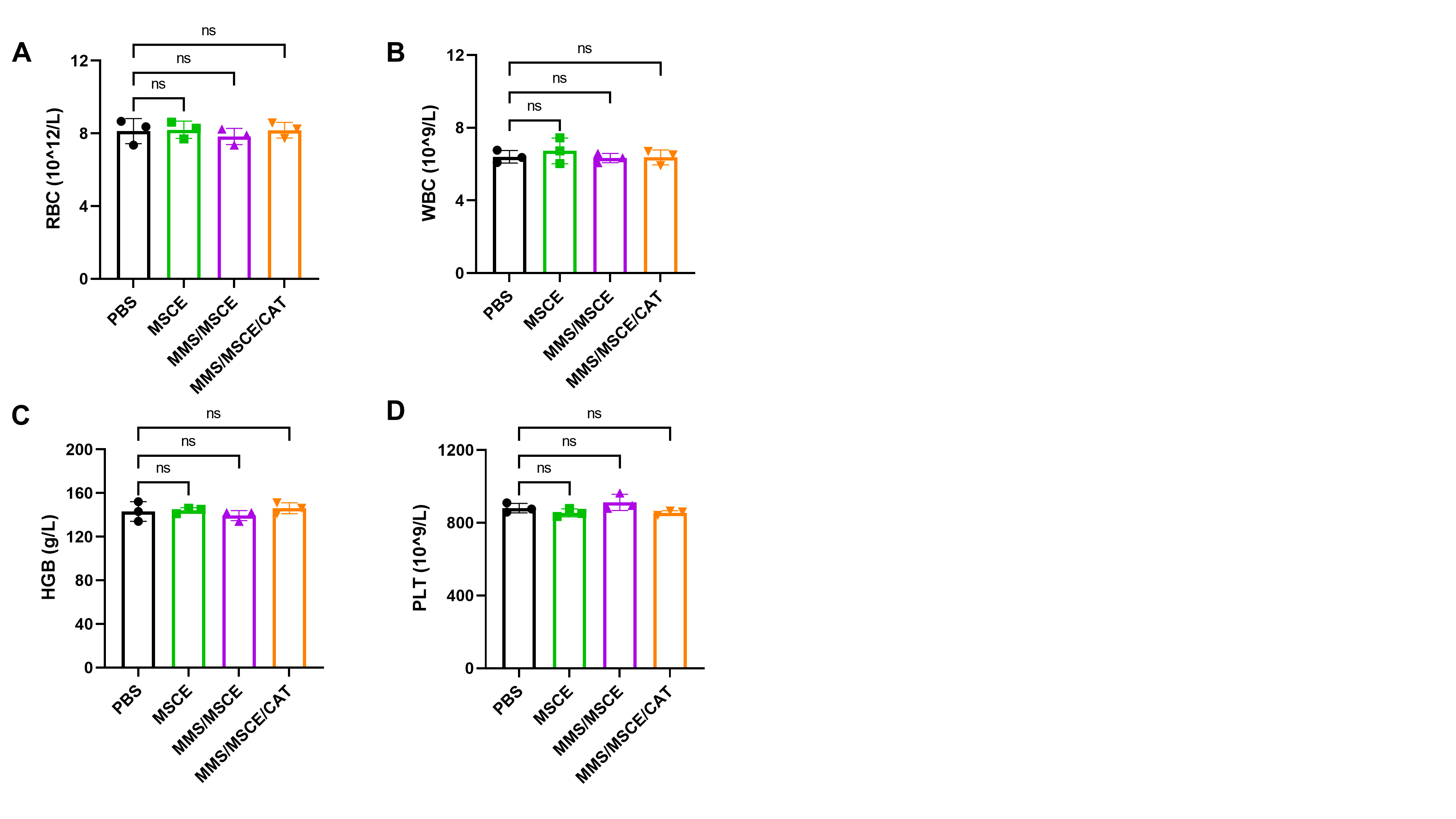


**Figure S21** Quantification of typical hematological parameters at 24 h after treatment with different samples in rats. The blood levels of (A) RBC, (B) WBC, (C) HGB and (D) PLT. Data are mean ± SD (*n* = 3).


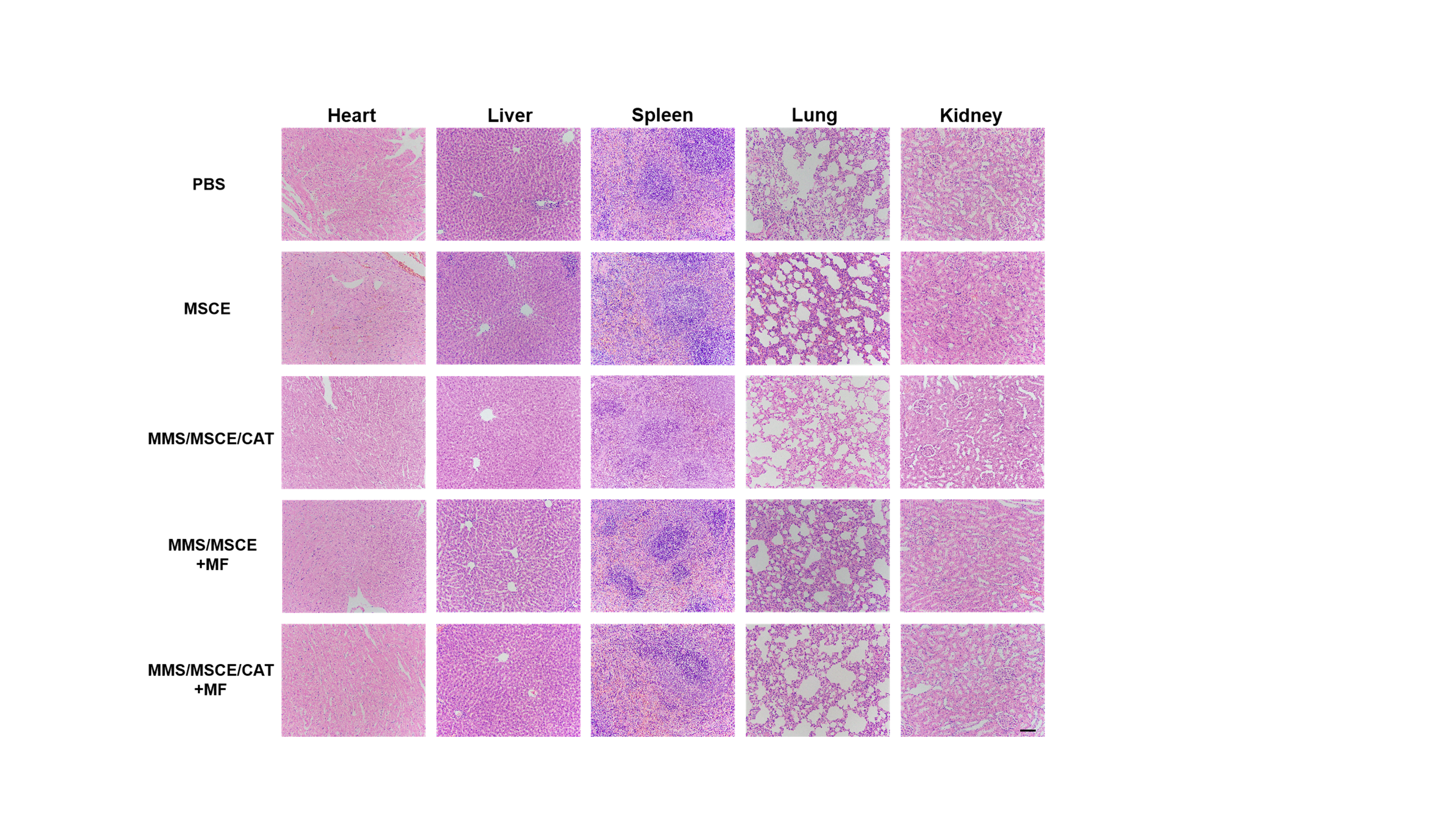


**Figure S22** H&E-stained pathological sections of typical major organs excised from rats at day 15 after treatment with different samples. Scale bar = 100 µm.

**Table S1** Summary of previous nanotherapies for AAA treatment.

| Year | Materials | Loaded drug | Targeting modalities | Therapy | Ref. |
| --- | --- | --- | --- | --- | --- |
| 2013 | DOX-PLGA NPs | Doxycycline (DOX) | Passive targeting effect | MMP activity↓(in vitro);  Elastin regenerative repair↑(in vitro) | ^[5]^ |
| 2013 | HA-o-NPs | HA-o | Passive targeting effect | Elastin regenerative repair↑(in vitro) | ^[6]^ |
| 2015 | EL-NP-BB94 | MMP inhibitor batimastat  (BB-94) | Antigen-antibody binding: conjugated with anti-elastin antibody | MMP activity↓;  Elastin degradation↓;  Calcification↓;  Aortic expansion↓(269% in control vs 40% in EL-NP-BB94) | ^[7]^ |
| 2016 | TGF-β1- and DOX-loaded PLGA NPs | DOX，  TGF-β1 | Passive targeting effect | MMP activity↓(in vitro);  Elastin regenerative repair↑(in vitro) | ^[8]^ |
| 2016 | PEG-b-PBLG NPs | Rapamycin (RAP) | Passive targeting effect | MMP activity↓;  Inflammatory responses↓ | ^[4]^ |
| 2016 | EL-PGG-NP | Pentagalloyl glucose (PGG) | Antigen-antibody binding: conjugated with anti-elastin antibody | MMP activity↓;  Elastin degradation↓;  Calcification↓;  Inflammatory responses↓;  Aortic expansion↓ | ^[9]^ |
| 2017 | DOX-SPION NPs | DOX | Magnetic field navigation | MMP activity↓(in vitro);  Elastin regenerative repair↑(in vitro) | ^[10]^ |
| 2017 | Cathepsin K pAb-conjugated DOX-SMPs | DOX | Antigen-antibody binding: conjugated with cathepsin K antibodies | MMP activity↓(in vitro);  Elastin regenerative repair↑(in vitro) | ^[11]^ |
| 2018 | CROR NP | RAP | ROS response;  Antigen-antibody binding: decoration with a peptide ligand cRGDfK;  Membrane modification: macrophage cell membrane | Calcification↓;  ROS-mediated oxidative stress↓;  Apoptosis↓;  Aortic expansion↓ | ^[12]^ |
| 2018 | PEG-PLGA NP | DOX | Passive targeting effect | MMP activity↓(in vitro);  Elastin regenerative repair↑(in vitro) | ^[13]^ |
| 2021 | pCDNA3.1-VS-1 NP | N-terminal fragment of chromogranin A Vasostatin-1 (VS-1) | Passive targeting effect | Aortic expansion↓ | ^[14]^ |
| 2021 | PGG-NPs | PGG | Antigen-antibody binding: conjugated with elastin antibody | Inflammatory responses↓;  Aortic expansion↓;  Elastin regenerative repair↑ | ^[15]^ |
| 2024 | ALN-decorated LaCD NP | Alendronate (ALN) | Calcification-targeting moiety | Elastin degradation↓;  Calcification↓;  Inflammatory responses↓;  Aortic expansion↓ | ^[16]^ |

[1] C. Thery, S. Amigorena, G. Raposo, A. Clayton, *Curr Protoc Cell Biol* **2006**, *Chapter 3*, Unit 3 22.

[2] S. Anidjar, J. L. Salzmann, D. Gentric, P. Lagneau, J. P. Camilleri, J. B. Michel, *Circulation* **1990**, *82*, 973-981.

[3] W. H. Huang, H. J. Liu, Y. C. Pan, X. Y. Wang, H. W. Yang, D. J. Wang, J. Lin, H. Zhang, *Journal of Cardiothoracic Surgery* **2023**, *18*.

[4] T. Shirasu, H. Koyama, Y. Miura, K. Hoshina, K. Kataoka, T. Watanabe, *PLoS One* **2016**, *11*, e0157813.

[5] B. Sivaraman, A. Ramamurthi, *Acta Biomater* **2013**, *9*, 6511-6525.

[6] A. Sylvester, B. Sivaraman, P. Deb, A. Ramamurthi, *Acta Biomater* **2013**, *9*, 9292-9302.

[7] N. Nosoudi, P. Nahar-Gohad, A. Sinha, A. Chowdhury, P. Gerard, C. G. Carsten, B. H. Gray, N. R. Vyavahare, *Circ Res* **2015**, *117*, e80-89.

[8] L. Venkataraman, B. Sivaraman, P. Vaidya, A. Ramamurthi, *J Tissue Eng Regen Med* **2016**, *10*, 1041-1056.

[9] N. Nosoudi, A. Chowdhury, S. Siclari, V. Parasaram, S. Karamched, N. Vyavahare, *J Cardiovasc Transl Res* **2016**, *9*, 445-455.

[10] B. Sivaraman, G. Swaminathan, L. Moore, J. Fox, D. Seshadri, S. Dahal, I. Stoilov, M. Zborowski, R. Mecham, A. Ramamurthi, *Acta Biomater* **2017**, *52*, 171-186.

[11] B. Jennewine, J. Fox, A. Ramamurthi, *Acta Biomater* **2017**, *52*, 60-73.

[12] J. Cheng, R. Zhang, C. Li, H. Tao, Y. Dou, Y. Wang, H. Hu, J. Zhang, *J Am Coll Cardiol* **2018**, *72*, 2591-2605.

[13] A. Camardo, D. Seshadri, T. Broekelmann, R. Mecham, A. Ramamurthi, *Drug Deliv Transl Res* **2018**, *8*, 964-984.

[14] P. Wang, W. Wang, X. Peng, F. Ruan, S. Yang, *Bioengineered* **2021**, *12*, 11018-11029.

[15] X. Wang, V. Parasaram, S. Dhital, N. Nosoudi, S. Hasanain, B. A. Lane, S. M. Lessner, J. F. Eberth, N. R. Vyavahare, *Sci Rep* **2021**, *11*, 8584.

[16] K. Hu, L. Zhong, W. Lin, G. Zhao, W. Pu, Z. Feng, M. Zhou, J. Ding, J. Zhang, *ACS Nano* **2024**, *18*, 6650-6672.
